# Anti-PD-1 blockade reverses low-intensity electric stimulation-driven pancreatic cancer progression

**DOI:** 10.3389/fimmu.2026.1793161

**Published:** 2026-05-19

**Authors:** Lingmin Jiang, Xiyuan Li, Dejun Zeng, Pu Xi, Zehui Yao, Qi Zhu, Shaopu Lian, Shengping Li, Chaobin He

**Affiliations:** 1 Department of Pancreatobiliary Surgery, State Key Laboratory of Oncology in South China, Guangdong Provincial Clinical Research Center for Cancer, Collaborative Innovation Center for Cancer Medicine, Sun Yat-sen University Cancer Center, Guangzhou, Guangdong, China; 2 Department of Head and Neck Surgery, State Key Laboratory of Oncology in South China, Guangdong Provincial Clinical Research Center for Cancer, Sun Yat-sen University Cancer Center, Guangzhou, Guangdong, China; 3 Department of General Surgery, Pingshan District Central Hospital of Shenzhen, Shenzhen, China; 4 Department of Head Neck and Thyroid, The Affiliated Cancer Hospital of Zhengzhou University and Henan Cancer Hospital, Zhengzhou, China

**Keywords:** anti-PD-1 blockade, CD8+ T cell, irreversible electroporation (IRE), low-intensity electric stimulation (LIES), pancreatic ductal adenocarcinoma (PDAC)

## Abstract

**Background:**

Irreversible electroporation (IRE) represents a potential therapeutic approach for pancreatic ductal adenocarcinoma (PDAC). However, differences in tumor size and shape can lead to an uneven distribution of the electric field, causing certain cells to receive only low-intensity electric stimulation (LIES). This study aimed to explore the tumor immune microenvironment following LIES and evaluate the synergistic antitumor effects of LIES combined with anti-PD-1 blockade.

**Methods:**

Orthotopic PDAC models in mice were treated with LIES with or without anti-PD-1 blockade. *In vitro* assays under varying electric fields assessed tumor cell viability, migration, and epithelial-mesenchymal transition (EMT). Tumor immune microenvironment remodeling was comprehensively profiled using flow cytometry, multicolor immunofluorescence, and single-cell RNA sequencing (scRNA-seq). The underlying mechanism was further investigated via bulk RNA sequencing and validated *in vitro*.

**Results:**

Orthotopic PDAC models were established and utilized for the first time to evaluate the immune modulation and macrophage polarization effects of LIES. Cell-based assays were conducted to assess the impact of varying field strengths on cell proliferation and migration. Anti-PD-1 blockade reversed the tumor growth and systemic antitumor responses following the LIES. Flow cytometry, multicolor immunofluorescence, and scRNA-seq revealed that combination therapy amplified CD8^+^ T cell activation. Further bulk RNA-seq analysis revealed that LIES activated the JAK2-STAT3 pathway, inducing expression of PD-L1 in PDAC cells.

**Conclusions:**

Anti-PD-1 blockade can reverse LIES-driven tumor progression by upregulating CD8^+^ T cell expression. The integration of anti-PD-1 blockade with IRE potentially overcomes the limitations of incomplete ablation within the LIES, thereby improving oncological outcomes.

## 1 Introduction

Pancreatic ductal adenocarcinoma (PDAC) is indeed one of the most aggressive and deadly forms of cancer. The 5-year survival rate for PDAC patients is alarmingly low, typically under 13% (1). Only about 15% of PDAC patients are eligible for surgical resection at the time of diagnosis. Unfortunately, the majority of PDAC patients, approximately 85%, present with locally advanced or metastatic disease, making them ineligible for surgery (2). At the time of diagnosis, around 35% of PDAC were found to present with locally advanced pancreatic cancer (LAPC), as determined by anatomical criteria observed through radiological imaging (3). Given that the mortality of patients with LAPC is largely due to tumor progression in its original location rather than its spread to distant sites, local ablative therapies emerge as promising therapeutic options (4).

Local ablative therapies have become significant adjunctive treatments for managing LAPC. Irreversible electroporation (IRE), a nonthermal ablative technique, permeabilizes tumor cell membranes with brief, high-voltage electrical pulses (5). This approach mitigates issues such as the heat sink effect and minimizes thermal damage to nearby tissues, thereby preserving crucial structures like blood vessels, bile ducts, and pancreatic ducts within the ablation zone (6). IRE provides a safer alternative for treating tumors located near these vital structures, making it a promising option for treating LAPC (7, 8). However, the recurrence observed in some PDAC patients following IRE treatment raises concerns about the impact of the treatment on the tumor microenvironment (TME). The effectiveness of IRE depends on the accurate delivery of electrical pulses, which can be influenced by factors such as the shape and size of the tumor, electrode placement, and the electrical properties of the tissues surrounding the tumor (9). Given the aggressive and uneven nature of PDAC, certain regions of the tumor might receive inadequate field intensity, resulting in incomplete ablation (10). This phenomenon, known as reversible electroporation (RE), can inadvertently promote tumor growth (11, 12). Zhao et al. reported that insufficient radiofrequency ablation (iRFA) promotes proliferation of residual hepatocellular carcinoma (13). A study from Su et al. showed that iRFA promotes hepatocellular carcinoma metastasis through an N6-methyladenosine mRNA methylation-dependent mechanism (14). Electric field intensities below 680 V/cm primarily induce RE, a phenomenon about low-intensity electric stimulation (LIES) (12, 15, 16). However, the local environment after LIES was unknown. As a result, it is crucial to explore how LIES affects the tumor’s local environment, as this may contribute to the observed recurrences. To enhance therapeutic efficacy, it is essential to identify treatments that can reverse the tumor microenvironment associated with recurrence and to combine therapeutic strategies to optimize treatment outcomes and improve patient prognosis. Immune checkpoint blockade is emerging as an effective cancer treatment, yielding durable responses in various tumor types (17–19). However, its efficacy in treating PDAC is hindered by the cancer’s immunosuppressive stroma (20). PDAC is marked by a dense fibrotic stroma that can physically block cytotoxic T cells from reaching tumor cells. Furthermore, the immunosuppressive environment within the stroma can suppress the activity of infiltrating T cells. Previous studies showed that anti-PD-1 blockade combined with IRE could improve the efficacy in PDAC. Some studies have examined the immune effects of IRE (21, 22). In pancreatic cancer, the combination of IRE with PD-1 blockade has been shown to promote the release of ATP and HMGB1, which, in turn, activate antigen presentation and generate cytotoxic CD8^+^ T cell responses (15). Another study discovered that cDC1-regulated antigen presentation plays a crucial role in activating cytotoxic CD8^+^ T cells following IRE treatment. Enhancing the cDC1-CD8^+^ T cell immune cycle significantly improved the effectiveness of PD-L1 blockade therapy in pancreatic cancer models (23). Wu et al. reported that IRE enhances the anti-tumor immune response in pancreatic cancer by activating the cDC2-CD4^+^ T-NK axis. When combined with PD-L1 blockade, this approach augments T cell and natural killer (NK) cell activity, suggesting that dual-target blockade of PD-L1/IL-6, alongside ablation therapy, could serve as a novel therapeutic strategy for MHC-I-deficient PDAC (24). Neither of these studies investigated the inflammatory environment following RE. The nature of the tumor microenvironment after LIES treatment therefore remains uncertain. Consequently, it is unknown whether immunotherapy could reverse the immune state following LIES-driven PDAC progression.

Epithelial-mesenchymal transition (EMT) is a pivotal mechanism underlying tumor invasion and metastasis, warranting focused attention. EMT facilitates the transformation of tumor cells from an epithelial phenotype, which enhances migratory, invasive, and anti-apoptotic properties. This transition plays a crucial role in the distant dissemination of tumors. Studies have shown that stress factors within the TME, such as radiotherapy and inflammation, can activate signaling pathways that induce the occurrence of EMT (25). However, the precise mechanisms through which LIES-induced senescence and EMT contribute to the diverse patterns of disease recurrence remain unclear. Understanding these processes is essential for developing strategies to mitigate tumor recurrence and improve therapeutic outcomes. Based on these analyses, we hypothesized that LIES can boost the efficacy of anti-PD-1 blockade therapy in PDAC by stimulating the immune system. This study revealed that LIES on its own did not induce cytotoxic effects but instead promoted cellular migration, and LIES could promote the expression of PD-L1. When combined with the anti-PD-1 blockade, it could reverse the infiltration of immune cells.

## 2 Materials and methods

### 2.1 Animal models

The animal experiments were conducted between August 2024 and July 2025 at the Sun Yat-sen University Cancer Center Laboratory Animal Center. The wild-type C57BL/6 mice were acquired from the Sun Yat-sen University Cancer Center Laboratory Animal Center. All mice were housed under specific pathogen-free conditions at the Sun Yat-sen University Cancer Center and performed in strict accordance with the guidelines of the Laboratory Monitoring Committee of Guangdong Province, China. Experiments were conducted using 6-week-old mice. All mice procedures were performed in strict accordance with the guidelines of Sun Yat-sen University. Mice were anesthetized with isoflurane and euthanized by carbon dioxide inhalation under deep anesthesia at the indicated time points for tumor harvesting or after the appearance of a tumor with a diameter greater than 1.5 cm in any group. All efforts were made to minimize animal suffering. An orthotopic pancreatic tumor model was established by injecting 1 × 10^6^ Pan02-luc cells into the pancreas. Mice were anesthetized, and a midline incision was made to expose the pancreas, where Pan02-luc cells in 100 μl Matrigel (Corning, 356231) were injected beneath the pancreatic capsule. The incision was then closed with sutures. Three weeks later, the mice were randomly assigned into the following groups (n = 5 per group) (1): untreated control, (2) LIES only, (3) anti-PD-1 blockade only, (4) LIES+anti-PD-1 blockade, (5) high-intensity electric stimulation (HIES) only, (6) HIES+ anti-PD-1 blockade (N = 30). The first group received intraperitoneal phosphate-buffered saline (PBS) injections three times per week as a placebo control. The second group underwent low-field electrical stimulation once. The third group was administered intraperitoneal injections of anti-PD-1 blockade three times per week. The fourth group received a combination of low-field electrical stimulation once and anti-PD-1 blockade injections. The fifth group underwent high-field electrical stimulation once, and the sixth group received a combination of high-field electrical stimulation once and anti-PD-1 blockade injections. The anti-PD-1 blockade (BE0146, BioXcell) was administered at a dose of 100 μg per mice through intraperitoneal injection on day 22, while LIES or HIES ablation was performed using an open laparotomy, as previously described (26). This was an exploratory efficacy study; the group size was selected based on prior studies (26). Each treatment cycle lasted 14 days, with LIES and HIES being conducted only once during the initial cycle. Tumor volume was measured using the Xenogen IVIS Spectrum (PerkinElmer). For imaging, D-Luciferin sodium salt at 100 mg/kg was given through intraperitoneal injection. The dimensions of the tumors, including length and width, were assessed, and the tumor volume was determined using the formula: (length × width^2^)/2. Finally, the mice were sacrificed for postmortem analysis at the indicated time points. The survival rates of the mice were also documented.

### 2.2 Cell culture

The BxPC-3 human PDAC cell line and Pan02 murine PDAC cell line were sourced from the Cell Bank of the Chinese Academy of Sciences in Shanghai, China. The cells were cultured at 37 °C in a 5% CO_2_ humidified incubator, utilizing either Rosewell Park Memorial Institute 1640 medium (RPMI 1640, R8758, Gibco) or Dulbecco’s modified Eagle medium (DMEM, C11995500BT, Gibco). The Pan02-luc cell line, which expresses a fusion gene of firefly luciferase and green fluorescent protein (GFP), was established through pLenti-CMV-EGFP-linker-Luc-PGK-Puro lentivirus (OBIO) and then sorted by a MoFlo High-Performance Cell sorter (Bec,kman Coulter, USA). All of these cell lines were enriched with 10% heat-inactivated fetal bovine serum (FBS, A5669701, Gibco) and 1% Penicillin-Streptomycin (15140122, ThermoFisher).

### 2.3 Electroporation

Electric pulses were generated using the ECM 830 electroporator from BTX Harvard Apparatus, Holliston, MA. Electroporation of cells *in vitro* or of orthotopic tumors *in vivo* was carried out as detailed in our previous study (27). To systematically investigate the biological effects of varying electrical intensities, a gradient of electric field strengths was established, including 100, 300, 750, 1000, and 1500 V/cm. Based on our preliminary findings and clinical relevance, 300 V/cm was formally defined as LIES to simulate the sublethal peripheral zone, while 1500 V/cm was defined as HIES to represent the effective ablation zone. For the *in vitro* experiments, a 400μL cell suspension containing 1 × 10^6^ cells, after digestion and counting, was placed in an electroporation cuvette (1652088; BTX, Holliston, MA, USA) situated between two aluminum electrodes with a 4-mm gap. The cell suspension was in direct contact with the plate electrodes and underwent electroporation at room temperature with the following parameters: a voltage range of 40-600V, a pulse duration of 100μs, a pulse frequency of 1 Hz, and up to 20 pulses, as needed. Specifically, 120V was applied for the LIES (300 V/cm) group and 600V for the HIES (1500 V/cm) group. For the *in vivo* experiments, a two-needle array electrode (BTX item #45-0168, BTX Harvard Apparatus, Holliston, MA) with a 5-mm inter-needle spacing was used. The electrode was inserted into the tumor sequentially along the X-, Y-, and Z-axes to ensure effective electroporation. The electroporation parameters were set as follows: a voltage range of 150-750V, a pulse duration of 100 μs, a pulse frequency of 1Hz, and a total of 80 pulses. Specifically, 150V was used for the LIES (300 V/cm) group and 750V for the HIES (1500 V/cm) group. Five animals were used for each experimental setting *in vivo* experiments, and three independent repetitions were performed for each experiment.

### 2.4 Cell viability assay and flat plate clone formation

Cell viability was evaluated using the Cell Counting Kit-8 (CCK-8) assay (HYC500, HUAYUN). For the assay, triturated cells were seeded into 96-well flat-bottom plates at a density of 2 × 10^3^ cells per well, with each well containing 100 μL of conditioned medium. Afterward, 10μL of CCK-8 solution was added to each well. Following a 2-hour incubation, absorbance was measured at 450nm to determine cell viability.

Following electroporation treatment, 500 cells were seeded into each well of a 6-well plate and cultured for 14 days, with the medium being refreshed every 3 days. The cells were then fixed with 4% formaldehyde solution and stained with crystal violet (E607309-0100, Sangon Biotech, Shanghai, China). Subsequently, colonies were counted and analyzed.

### 2.5 Transwell migration assay and cell scratch

For the transwell migration assay, chambers with 8μm pores (353097, Corning) were used. The lower chamber was supplemented with medium containing 20% FBS. After treatment, cells were counted to 1 × 10^4^ and placed into the upper chamber. Following a 24-hour incubation, cancer cells that had migrated through the membrane and adhered to the underside were fixed with paraformaldehyde and stained with crystal violet. Migrated cells were then imaged and counted under a 20× objective lens. Statistical analysis was performed based on three independent experiments, averaging over five microscopic fields per experiment.

For the scratch wound assay, tumor cells from different treatment groups were adjusted to a concentration of 1 × 10^5^ and seeded into 12-well plates. A sterile 20μL pipette tip was used to create a wound gap by scratching the cell layer. The migration rate was determined by measuring the wound width at 0 and 24 hours, and calculating the percentage of wound closure relative to the initial wound length.

### 2.6 Western blotting

Cells were lysed using the RIPA buffer, then subjected to sodium dodecyl sulfate-polyacrylamide gel electrophoresis (SDS-PAGE) and subsequently transferred onto polyvinylidene difluoride (PVDF) membranes (Millipore, Billerica, MA, USA). A prestained protein marker (MP102-02, Vazyme) was run in parallel to estimate molecular weights. These membranes were incubated overnight at 4 °C with the primary antibodies, as detailed in **Supplementary Table 1**. After three washes with Tris-buffered saline containing 0.05% Tween-20, membranes were incubated for 1 hour at room temperature with either anti-rabbit or anti-mouse secondary antibodies. Protein bands were visualized by using an ECL detection kit (4AW011, Biotech Co., Ltd). Images were prepared using the ChemiDoc XRS + system (BioRad, China), and quantification analyses were performed by Bio-Rad Image Lab software (BioRad, China).

### 2.7 Immunohistochemistry and immunohistofluorescence

Tumor sections (4μm) were deparaffinized in xylene, rehydrated through a graded ethanol series, and washed with phosphate-buffered saline. Antigen retrieval was performed by pressure cooking the sections for 3 minutes in EDTA buffer (pH 8.0), followed by blocking in PBS containing 3% bovine serum albumin (BSA) at room temperature for 30 minutes. Subsequently, the sections were incubated with monoclonal antibodies or isotype controls overnight at 4 °C, as detailed in **Supplementary Table 1**.

For IHC, the 3,3'-diaminobenzidine (DAB) system was utilized to visualize staining. Tissue sections were washed with PBS containing 0.1% Tween-20. Following further washing, they were incubated with biotinylated goat anti-rabbit IgG for 2 hours at room temperature. Immunostaining was visualized using a streptavidin-horseradish peroxidase (HRP) conjugate and diaminobenzidine. Finally, the sections were counterstained with hematoxylin and examined under a bright-field microscope at 40× and 200× magnification.

### 2.8 Flow cytometry

Mice bearing orthotopic tumors were euthanized 14 days post-IRE treatment, and tumors were harvested and dissociated using a mouse tumor dissociation kit, following the manufacturer’s instructions (Miltenyi Biotec, Germany). The resulting single-cell suspension was filtered through a 70-μm cell mesh and resuspended in PBS containing 2% FBS. Similarly, spleens were triturated, filtered through a 40-μm cell mesh, and resuspended in PBS with 2% FBS. For surface staining, the cell samples were incubated with specific antibodies for 30 minutes on ice, in the dark.

For intracellular staining, cells were fixed using a fixation/permeabilization concentrate (Invitrogen), mixed with a fixation/permeabilization diluent (Invitrogen) in a 1:3 ratio, and incubated for 40 minutes at 37 °C, followed by two washes with a diluted 1 × permeabilization buffer (10 ×, BioLegend). For cytokine staining, T cells were cultured in a medium containing a cell stimulation cocktail (500 ×, Invitrogen) at 37 °C for 4 hours. Subsequently, cells were incubated with the specified antibodies for 30 minutes at 37 °C in the dark. Flow cytometry analysis was conducted using the Cytek Aurora system (Cytek Biosciences), and the data were processed with FlowJo software. The fluorescent dye-labeled antibodies used in this study are summarized in **Supplementary Table 1**.

In our *in vitro* studies focusing on tumor cells, BxPC-3 and Pan02 cells were first collected, and their concentration was adjusted to 1 × 10^6^ cells after electroporation. The cells were incubated with 10 μL phycoerythrin-conjugated anti-human PD-L1 (CD274, 329705, BioLegend) at room temperature for 5 minutes in the dark. After staining, the cells were subsequently analyzed using a flow cytometer.

### 2.9 qRT-PCR analysis

Total RNA was isolated from cultured cells utilizing the RNA Quick Purification Kit (400-100, GOODNIE) in strict accordance with the provided manufacturer’s protocol. Subsequently, the extracted RNA was converted into complementary DNA (cDNA) using the PrimeScript™ RT Master Mix (Takara, Japan), again adhering to the manufacturer’s guidelines. Quantitative reverse transcription polymerase chain reaction (qRT-PCR) was conducted in triplicate employing SYBR Green as the fluorescent dye, on a Roche LightCycler 480 Real-Time PCR System. The specific primers utilized for the qRT-PCR analysis are detailed in **Supplementary Table 2**.

### 2.10 Tissue dissociation and cell purification

Tissue samples were transported on ice in Dulbecco’s DMEM supplemented with 1mM protease inhibitor (CW2200S, CWBIO) to preserve cell viability. Samples were subjected to three washes with PBS and subsequently minced on ice. For tissue digestion, we employed an enzyme mixture comprising 1mg/mL Trypsin Inhibitor (T6522, Sigma-Aldrich) and 1 unit/mL DNase I (M0303S, New England Biolabs), all dissolved in PBS with 5% FBS.

The resulting cell suspension was filtered through a 40-μm nylon cell strainer (model 352340, Falcon), and erythrocytes were removed using a red blood cell lysis buffer (1966634, Invitrogen). Cell viability was assessed with 0.4% trypan blue solution (T10282, Invitrogen). Finally, cells were resuspended in PBS containing 0.04% BSA to achieve a concentration of approximately 1 × 10^6^ cells per milliliter, suitable for single-cell RNA sequencing (scRNA-seq) applications.

### 2.11 Single-cell RNA sequencing and initial data processing

Orthotopic pancreatic tumor models were established in C57BL/6 mice using Pan02 cells. One week following treatment, mice from each group (control, LIES only, anti-PD-1 blockade only, LIES+anti-PD-1 blockade, HIES only, HIES+ anti-PD-1 blockade; n = 4 per group) were sacrificed. From these, three tumors with optimal morphology (uniform size, minimal necrosis, and intact capsule) were selected per group for scRNA-seq, resulting in 3 independent biological replicates per condition (18 tumors total). Tumors were gently rinsed to remove blood and trimmed to exclude non-tumor tissues. Clean samples were immediately frozen in liquid nitrogen, transferred to dry ice, and sent to a commercial provider for scRNA-seq and bioinformatic analysis.

Single-cell suspensions were prepared following the standardized protocol of the Chromium Single-Cell 3’ Reagent Kit, to capture a range of 5000 to 10000 cells per chip position (utilizing V2 chemistry). Subsequent steps, including library preparation, were meticulously performed in strict accordance with the manufacturer’s established guidelines. Libraries were sequenced using the Illumina HiSeq × Ten platform with a paired-end strategy, producing 150-nucleotide reads. The resulting data were analyzed utilizing the Cell Ranger 2.1.0 software suite, following recommended configurations. FASTQ files generated from the sequencing were aligned to the GRCm39 reference genome using the STAR alignment algorithm (28). The filtered feature-barcode matrices from Cell Ranger were imported into Seurat version 5.0.0 for downstream analysis. Cells were retained for further analysis if they met the following quality control criteria: number of detected genes between 200 and 6000, and mitochondrial gene content < 10%. Genes detected in fewer than three cells were excluded. To integrate data from the 18 independent biological replicates and mitigate potential batch effects, the Harmony algorithm was employed using the RunHarmony function with default parameters, using the first 30 principal components as input. This ensured that subsequent clustering was driven by biological states rather than technical artifacts.

### 2.12 Dimensionality reduction, clustering, and cell type annotation

To identify primary cell types, the Seurat package in R was employed. A set of highly variable genes was selected and used for conducting Principal Component Analysis (PCA). Clusters were visualized using Uniform Manifold Approximation and Projection (UMAP), based on the first ten principal components calculated via the RunUMAP function. To characterize the cellular identities within these clusters, established markers were referenced: Ptprc for immune cells, Pecam1 for endothelial cells, and Acta2 for stromal cells. To identify genes distinguishing clusters, the FindAllMarkers function of the Seurat package was applied to the normalized gene expression data using the Wilcoxon rank-sum test, requiring a log2 fold change (FC) > 0.25 and an adjusted p-value < 0.05.

To quantify tumor cell plasticity, EMT scores were calculated using the AddModuleScore function based on the MSigDB Hallmark EMT gene set. Differential expression across the six experimental groups was determined using the Wilcoxon rank-sum test, applying the same thresholds (log2FC > 0.25, adjusted p < 0.05). For comparisons involving more than two groups, pairwise tests were performed, and p-values were adjusted using the Bonferroni correction.

### 2.13 Bulk RNA sequencing

In summary, the process commenced with 500ng of purified RNA, possessing a high integrity score (RIN > 7), specifically targeting polyadenylated mRNA. This was followed by a sequence of procedures, including RNA fragmentation, cDNA synthesis using random primers as part of the NEBNext protocol, PCR-based indexing, and the selection and quantification of cDNA fragments using KAPA reagents from Roche. The prepared cDNA libraries were sequenced on the Illumina NovaSeq 6000 platform. For alignment and read quantification, the GRCm39 reference genome was used, applying methods previously described. This included the use of the STAR aligner (version 2.7.8) for mapping reads, featureCounts (version 1.6.4) for read counting, and Ensembl gene transcripts (version 104) for annotation. Differential gene expression analysis was conducted using the DESeq2 software package (version 1.30), allowing for rigorous evaluation of gene expression changes across samples.

### 2.14 Patients

A retrospective survival analysis was conducted on LAPC patients who underwent either IRE ablation alone or in combination with anti-PD-1 blockade therapy. The human study enrolled 108 LAPC patients at the Sun Yat-sen University Cancer Center between August 2015 and March 2024. Given the retrospective nature of this study, treatment allocation (IRE vs. IRE + anti-PD-1) was determined by clinical indications and patient preference, rather than randomization. Consequently, neither the clinicians nor the patients were blinded to the treatment assignments. The study was approved by the institutional review board of Sun Yat-sen University Cancer Center. Informed consent for participation in the study has been obtained. All patients were recruited based on uniform inclusion and exclusion criteria to ensure cohort comparability (**Supplementary Figure 5A**). All patients were routinely followed up after treatment.

### 2.15 Statistical analyses

Statistical analyses conducted in this study are detailed in the figure legends. Results are presented as mean ± standard deviation. Comparisons between two groups were performed using either unpaired or paired (for matched groups) two-tailed Student’s t-tests, or with the nonparametric Mann-Whitney U-test. Categorical variables and baseline characteristics were compared using the Chi-square test or Fisher’s exact test. Survival analyses, including overall survival (OS) and progression-free survival (PFS), were evaluated using the log-rank test. To address potential selection bias and confounding factors inherent in this retrospective cohort, multivariable Cox proportional hazards regression models were employed for statistical correction. Variables with a p-value < 0.10 in the univariable analysis were included in the multivariable model to identify independent prognostic factors. Correlations between parameters were assessed using Pearson’s correlation coefficient. All statistical tests were executed using GraphPad Prism 8 (GraphPad Software), Statistical Product and Service Solutions (SPSS version 25.0, IBM, Chicago, USA), and R software (version 4.1.1, R Foundation for Statistical Computing, Vienna, Austria). A p-value of less than 0.05 was considered indicative of statistical significance.

## 3 Results

### 3.1 Enhancement of cancer cell migration by LIES

**Figure 1:**
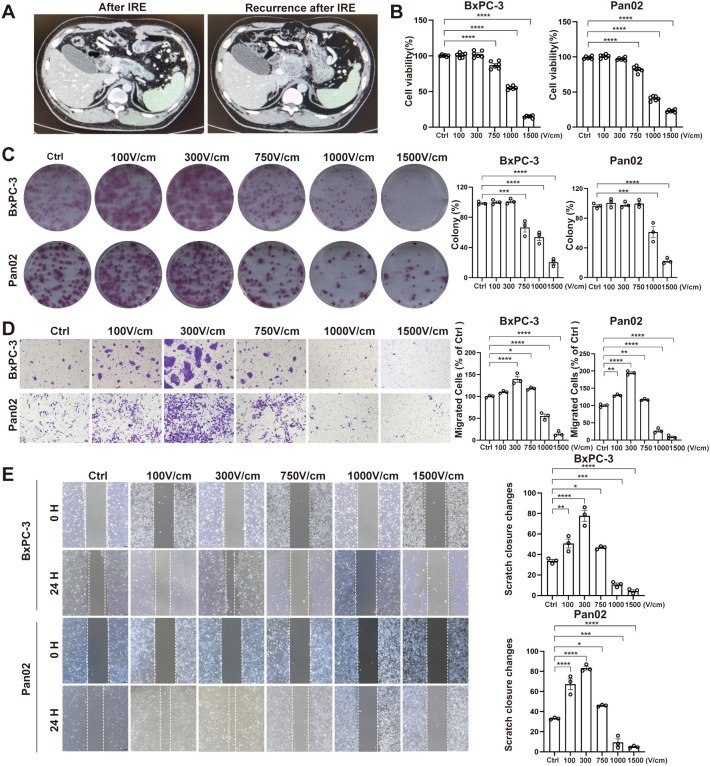
Low-intensity electric stimulation (LIES) enhance cell proliferation and migration without causing cytotoxicity. **(A)** The computed tomography scan images about recurrence following irreversible electroporation treatment. **(B, C)** The CCK8 and colony formation assay of BxPC-3 and Pan02 cells under different electric field strengths. **(D, E)** Representative images of transwell migration and scratch assays. LIES enhanced migration, while high-intensity fields inhibited it. Scale bar, 100 μm. Data in **(B–D)** are presented as mean ± SD (n = 3); *p < 0.05; **p < 0.01; ***p < 0.001; ****p < 0.0001, significant difference compared with the control (one-way ANOVA, Dunnett’s test).

In clinical practice, pose-IRE recurrence is a major factor influencing prognosis (**Figure 1A**). In an *in vitro* experiment, we evaluated the effects of various electric field intensities, ranging from 100V/cm to 1500V/cm, with intermediate levels at 300V/cm, 750V/cm, and 1000V/cm, on tumor cell viability and migration. The results indicated that field intensities of ≤ 750V/cm affect cell proliferation, with the majority of cells remaining viable (**Figures 1B, C**), as assessed using CCK 8 and colony formation assay. Migration assays, including scratch and transwell tests, revealed enhanced cell migration under LIES, while higher field strengths (≥1000V/cm) markedly inhibited migration (**Figures 1D, E**).

### 3.2 LIES upregulated PD-L1 expression and a shift toward a mesenchymal-like phenotype in tumor cells

**Figure 2:**
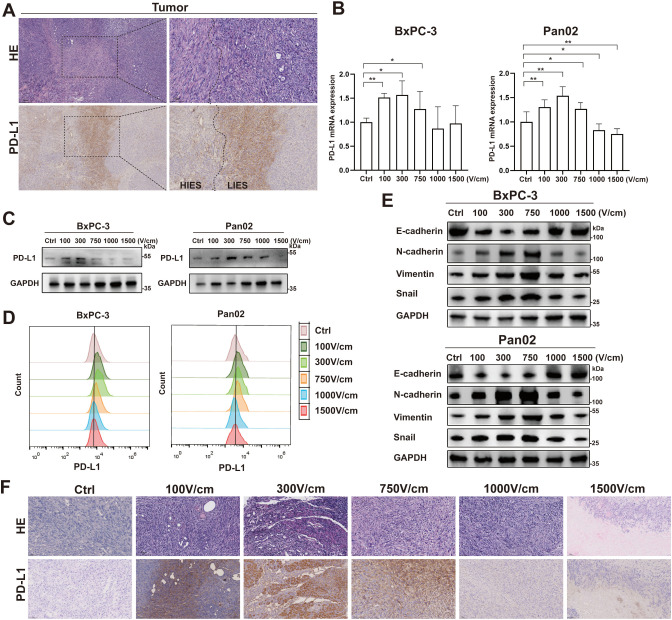
Low-intensity electric stimulation (LIES) upregulates PD-L1 expression and epithelial-mesenchymal transition (EMT) in tumor cells. **(A)** Representative hematoxylin and eosin (H&E) staining and PD-L1 immunohistochemical staining (IHC) of orthotopic pancreatic tumors after irreversible electroporation treatment, including areas of high-intensity electric stimulation and LIES treatment. **(B–D)** QRT-PCR **(B)**, western blot [WB, **(C)**], and flow cytometry **(D)** showing the expression of PD-L1 in BxPC-3 and Pan02 cells after varying electric field intensities. **(F)** H&E and PD-L1 IHC staining in orthotopic pancreatic tumor models under different electric field strengths. **(E)** WB analysis of EMT markers (N-cadherin, Vimentin, and Snail, and E-cadherin) in tumor cells after 24 hours under different electric field strengths. Low-magnification images, Scale bar, 250 μm; High-magnification images, Scale bar, 100 μm. Data in **(B)** are presented as mean ± SD (n = 3); *p < 0.05; **p < 0.01; ***p < 0.001; ****p < 0.0001, significant difference compared with the control (one-way ANOVA, Dunnett’s test).

The upregulation of PD-L1 expression in peripheral regions adjacent to HIES zones, corresponding to areas exposed to LIES, was confirmed by IHC staining (**Figure 2A**). To evaluate PD-L1 expression, BxPC-3 and Pan02 cells were exposed to varying electric field intensities. QPCR, WB, and flow cytometry analyses consistently revealed that LIES significantly upregulated PD-L1 expression (**Figures 2B–D**). In contrast, HIES did not result in a significant upregulation of PD-L1 expression. Furthermore, *in vivo* experiments confirmed the modulatory effect of electric field intensities on PD-L1 levels within tumor tissues (**Figure 2F**). Taken together, these results suggest that LIES specifically promotes PD-L1 expression. Additionally, WB analysis demonstrated that LIES was associated with a shift toward a mesenchymal-like phenotype, as evidenced by increased expression of Snail, N-cadherin, and vimentin, along with decreased levels of E-cadherin (**Figure 2E**).

### 3.3 Combined LIES and anti-PD-1 blockade treatment suppresses PD-L1 and expression of EMT-related markers

**Figure 3:**
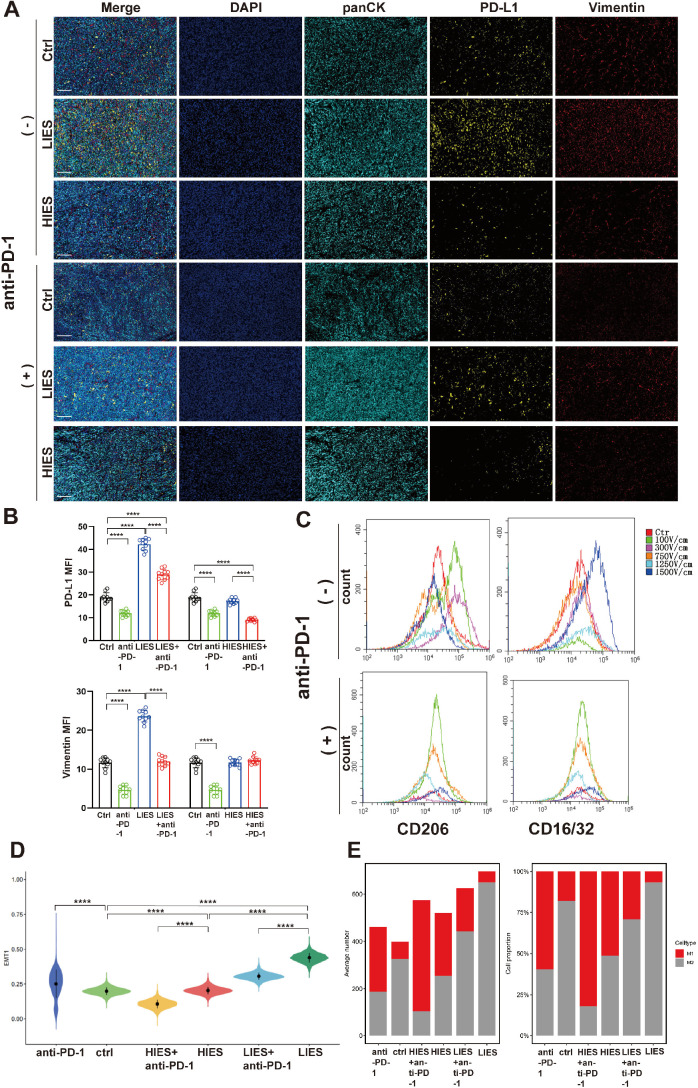
Combined low-intensity electric stimulation (LIES) and anti-PD-1 blockade treatment suppresses PD-L1 and epithelial-mesenchymal transition (EMT) expression. **(A)** Immunofluorescence (IHF) analysis in tumor sections from orthotopic pancreatic tumors. Nuclei were stained with DAPI (blue), tumor cells with panCK (cyan) and PD-L1 (yellow), and mesenchymal marker vimentin (red) fluorescence. Merged images were displayed in the rightmost column. Scale bar, 50 μm. Data in **(B)** are shown as relative PD-L1 and Vimentin expression. **(C)** Flow-cytometric quantification of CD206 and CD16^+^/32^+^ surface expression on macrophages after 24h co-culture with BxPC-3 and Pan02 cells pre-treated at varying electric field strengths. **(D)** The violin plot illustrated the distribution of EMT marker expression under different electric field strengths and anti-PD-1 blockade (anti-PD-1, ctrl, high-intensity electric stimulation [HIES] +anti-PD-1, HIES, LIES+anti-PD-1, LIES). **(E)** Single-cell RNA sequencing analysis of orthotopic tumors. UMAP analysis identified distinct clustering patterns of macrophages from orthotopic tumors. The bar graph showed the proportion of macrophages in each group. Data in **(B)** are presented as mean ± SD (n = 10). *p < 0.05; **p < 0.01; ***p < 0.001; ****p < 0.0001, significant difference compared with the control (one-way ANOVA, Dunnett’s test).

We also assessed PD-L1 expression and EMT *in vivo*. IHF revealed that LIES significantly upregulated PD-L1 and vimentin expression, while HIES failed to induce a significant increase in either PD-L1 or vimentin (**Figures 3A, B**). Following anti-PD-1 blockade, both PD-L1 and vimentin levels were markedly attenuated in the LIES group, indicating that neutralizing PD-L1 not only abrogates its immune checkpoint function but also accompanied by a shift away from the mesenchymal phenotype, thereby underscoring a mechanistic link between PD-L1 signaling and EMT-related plasticity. Additionally, flow cytometry analysis revealed that LIES significantly increased the proportion of M2 macrophages, as indicated by elevated CD206 expression (**Figure 3C**). In contrast, HIES treatment was associated with a gradual reduction in CD206 expression, suggesting a potential inhibitory effect on M2 macrophage polarization. Upon combination with anti-PD-1 blockade, CD206 expression was markedly decreased all field strength groups. IHF analysis further confirmed that LIES treatment was associated with a trend toward M2 macrophage polarization, as evidenced by the increased expression of CD206 (**Supplementary Figure 1A**). After anti-PD-1 blockade treatment, CD206 expression was markedly reduced (**Supplementary Figure 1B**). These findings suggest that anti-PD-1 treatment may reshape the immune microenvironment into a state less conducive to M2 polarization and mesenchymal features.

To further elucidate the molecular alterations induced by electric stimulation in tumor tissues, orthotopic tumor samples from three randomly selected mice per group were subjected to scRNA-seq. UMAP visualization revealed distinct clustering of cell populations (**Supplementary Figure 1C**, upper left panel), which were further annotated into major cell types, including epithelial cells, NK cells, T cells, macrophages, dendritic cells (DCs), stromal cells, and B cells (**Supplementary Figure 1C**, upper right panel). Cells from different treatment groups were distributed across clusters with some variation in cellular composition (**Supplementary Figure 1C**, lower panel). Marker gene expression analysis, summarized in the bubble plot, validated the cell type annotations, with epithelial cells showing high expression of T cells markers such as CD3, and macrophages expressing CD68 and CD74 (**Supplementary Figure 1D**). Importantly, differential analysis of EMT scores across groups revealed a significant upregulation of EMT-related gene expression in tumor cells exposed to LIES compared to control and HIES groups. After anti-PD-1 blockade treatment, EMT-associated transcriptional activation was suppressed in both the LIES and HIES groups (**Figure 3D**). To further validate these observations, single-cell RNA sequencing of tumor-infiltrating macrophages revealed a higher proportion of M2 subpopulations in the LIES group, while the HIES group exhibited a predominantly M1 phenotype. UMAP analysis of the single-cell data is shown in **Supplementary Figures 2A, B**. Notably, the addition of anti-PD-1 blockade to LIES-treated tumors was accompanied by a rebalancing of macrophage subsets toward an M1-like state, alongside a concomitant reduction in M2-like abundance (**Figure 3E**). These findings further demonstrated that LIES combined with anti-PD-1 blockade is associated with an attenuation of the mesenchymal signature.

### 3.4 Combined LIES and anti-PD-1 blockade treatment enhances CD8^+^ T cell infiltration

**Figure 4:**
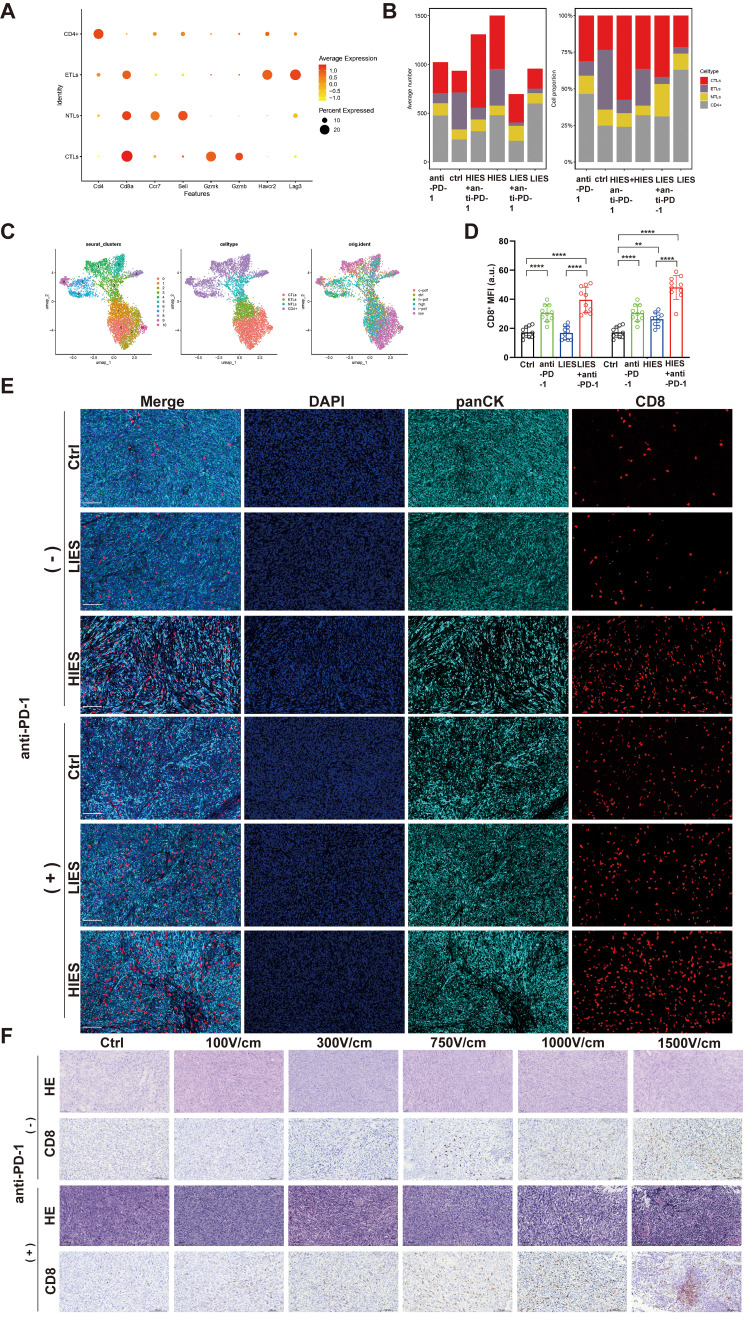
Combined low-intensity electric stimulation (LIES) and anti-PD-1 blockade treatment enhances CD8^+^ T cell infiltration. **(A)** Dot plot displayed the expression of key marker genes across different cell identities. Each dot represents a gene, with the size indicating the percentage of cells expressing the gene and the color representing the average expression level. **(B)** The bar graph showed the proportion of cytotoxic T lymphocytes (CTLs) and exhausted T lymphocytes (ETLs) in each group. **(C)** UMAP plots showed the clustering of single cells based on different annotation schemes: (left) Seurat clusters, (middle) distinct clustering patterns of immune cells, and (right) original sample identity. Cells are color-coded according to their assigned clusters, cell types, and sample identities. **(D, E)** Immunofluorescence (IHF) analysis in tumor sections from orthotopic pancreatic tumors. Nuclei were stained with DAPI (blue), tumor cells with panCK (cyan) and CD8^+^ (red) fluorescence. Merged images were displayed in the leftmost column. Scale bar, 50 μm. Data in **(D)** are shown as relative CD8^+^ expression. **(F)** Representative immunohistochemical staining of CD8^+^ in orthotopic tumor tissue sections under different electric field strengths, administered alone or in combination with anti-PD-1 blockade. Scale bar, 100 μm. Data in **(D)** are presented as mean ± SD (n = 10). *p < 0.05; **p < 0.01; ***p < 0.001; ****p < 0.0001, significant difference compared with the control or without anti-PD-1 blockade treatment (one-way ANOVA, Dunnett’s test).

To gain deeper insight into the mechanisms underlying IRE ablation and its potential synergistic effects with immunotherapy, we performed scRNA-seq on tumor-infiltrating immune cells. UMAP visualization revealed distinct clustering of cell populations (**Figure 4C**). In the scRNA-seq data, cytotoxic T lymphocytes (CTLs) exhibited high expression of CD8a and Gzmb, while exhausted T lymphocytes (ETLs) displayed elevated levels of immune checkpoint molecules such as Havr2 and Lag3 (**Figure 4A**). After IRE stimulation, a significant upregulation of CD8^+^ expression was observed in the HIES group, while the LIES group did not exhibit noticeable changes. Meanwhile, following anti-PD-1 blockade treatment, both the LIES and HIES groups exhibited higher proportions of CTLs compared to the control group (**Figure 4B**). Furthermore, IHF showed changes in CD8^+^ cells within the tumor microenvironment under various treatment conditions. CD8^+^ T cell expression was lower in the LIES and control groups compared to the HIES group. Moreover, a significant increase in CD8^+^ T cell infiltration was evident in the LIES combined with anti-PD-1 blockade group compared to the LIES group (**Figures 4D, E**). These findings suggest that LIES, especially when combined with anti-PD-1 blockade treatment, effectively attenuates the immunosuppressive microenvironment while promoting CD8^+^ T cell-mediated anti-tumor immunity.

To further analyze CD8^+^ T cell infiltration, IHC analysis demonstrated a progressive increase within tumor tissues as the electric field strength was elevated. In the absence of anti-PD-1 blockade, the HIES groups exhibited a marked enhancement in CD8^+^ T cell infiltration. When combined with anti-PD-1 blockade, all electric field strength groups showed further increases in intratumoral CD8^+^ T cell infiltration (**Figure 4F**). These findings indicate that electric field stimulation effectively promotes CD8^+^ T cell infiltration in the tumor microenvironment, and the addition of anti-PD-1 blockade can further potentiate this effect across different electric field intensities. This suggests a potential synergistic anti-tumor effect of combining electric field stimulation with immunotherapy.

In addition to assessing CD8^+^ T cell infiltration, we performed flow cytometry analysis to further investigate changes in the tumor immune microenvironment. Flow cytometry analysis revealed that, in the absence of anti-PD-1 blockade, both the proportion of mature DCs (CD80^+^ CD11c^+^) and effector memory T cells (CD62L^-^ CD44^+^) were relatively low in the control group (**Supplementary Figure 3A**). As the electric field strength increased, there was a gradual elevation in the percentages of mature DCs and effector memory T cells, while the proportion of central memory T cells (CD62L^+^ CD44^+^) remained largely unchanged. Notably, when combined with anti-PD-1 blockade, all groups exhibited significantly higher proportions of mature DCs and effector memory T cells compared to their counterparts without anti-PD-1 blockade, with the most pronounced synergistic effects observed at HIES (**Supplementary Figures 3C, D**). These findings indicated that electric field stimulation can substantially promote the maturation of DCs and the induction of effector memory T cells in the tumor microenvironment, thereby enhancing the memory and durability of anti-tumor immune responses. The addition of anti-PD-1 blockade further amplified these effects across all electric field intensities, particularly under HIES, suggesting a synergistic improvement in the tumor immune microenvironment by combining electric field stimulation with anti-PD-1 blockade. In contrast, analysis of the splenic immune cell populations demonstrated that varying electric field strengths and anti-PD-1 blockade, either alone or in combination, had no significant effect on the proportions of activated DCs, CD4^+^ T cells, CD8^+^ T cells, or effector memory T cells (**Supplementary Figure 4A**). However, further subset analysis revealed that upon anti-PD-1 blockade, the proportions of both effector and central memory T cells in the spleen increased in a field strength-dependent manner, with the greatest enhancement observed at HIES (**Supplementary Figures 3B, E, F**). This suggests that while electric field stimulation or anti-PD-1 blockade alone did not markedly alter systemic T cell activation, their combination can potentiate splenic memory T cell responses under HIES.

### 3.5 The combination of LIES and anti-PD-1 blockade therapy results in a marked reduction in tumor growth

Using an orthotopic murine model of PDAC, we evaluated the effects of LIES and HIES on tumor growth. Mice were randomized into six groups (1): untreated control, (2) LIES only, (3) anti-PD-1 blockade only, (4) LIES + anti-PD-1blockade, (5) HIES only, (6) HIES + anti-PD-1 blockade.

**Figure 5:**
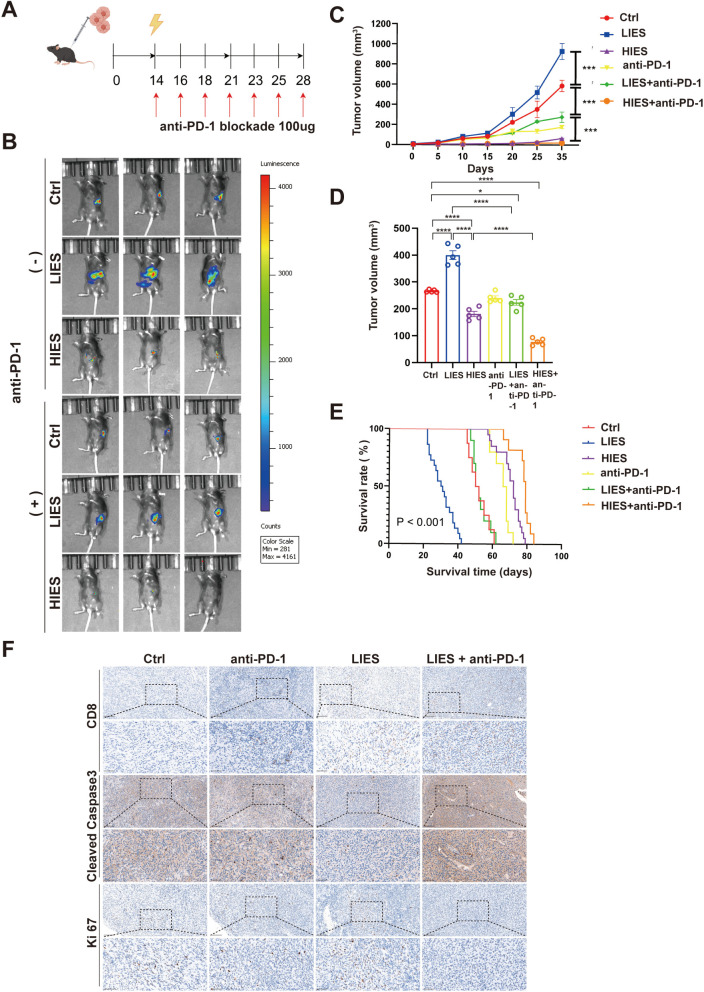
The combination of low-intensity electric stimulation (LIES) and anti-PD-1 blockade therapy results in a marked reduction in tumor growth. **(A)** The timeline and scheme of the experimental setup for orthotopic mice experiments. 1 × 10^6^ Pan02 cells were implanted into the pancreas. Treatment with electroporation at different electric field strengths (LIES: 300 V/cm, high-intensity electric stimulation [HIES]: 1500 V/cm) and each with or without concurrent anti-PD-1 blockade. Tumor size was measured every 5 days after treatment. **(B)** Representative *in vivo* bioluminescence imaging of tumor growth from mice receiving different electric field strengths and the corresponding regimens combined with anti-PD-1 blockade. **(C)** Tumor growth curves of subcutaneous tumors after treatment with different electric field intensities, administered alone or in combination with anti-PD-1 blockade. **(D)** Tumors weight of subcutaneous tumors after treatment with different electric field intensities, administered alone or in combination with anti-PD-1 blockade. **(E)** Survival analysis of mice under different conditions (n=15 per group). Statistical significance was assessed by log-rank test (P < 0.001). **(F)** Representative immunohistochemical staining of CD8^+^, Cleaved Caspase 3, and Ki 67 in orthotopic tumor tissue sections. Low-magnification images, Scale bar, 100 μm; High-magnification images, Scale bar, 50 μm. Data in **(C, D)** are expressed as mean ± SD (n = 5). *p < 0.05; ***p < 0.001; ****p < 0.0001, significant difference compared with the control (two-way ANOVA, Tukey’s test).

LIES and HIES were subsequently applied in a tumor mouse model, and tumor volumes were assessed following treatment to evaluate therapeutic efficacy (**Figure 5A**). Notably, LIES failed to inhibit tumor growth; on the contrary, it resulted in enhanced tumor progression (**Figure 5B**). In contrast, HIES significantly reduced tumor size. In contrast to the tumor-promoting effect of LIES monotherapy, the combination of LIES and anti-PD-1 blockade abolished the pro-tumorigenic outcome and effectively suppressed tumor growth. Tumors in the HIES combined with anti-PD-1 blockade group were smaller than those in either the HIES or anti-PD-1 monotherapy groups (**Figures 5C, D**). Regarding OS, the LIES exhibited the shortest OS among all groups; however, the addition of anti-PD-1 blockade demonstrated improved survival compared to LIES alone (**Figure 5E**). The HIES combined with anti-PD-1 blockade also showed favorable OS, either HIES or anti-PD-1 monotherapy alone.

To further investigate the therapeutic efficacy of LIES and anti-PD-1 blockade treatment, IHC analysis was conducted to examine apoptosis, proliferation, and CD8^+^ T cells in tumor tissues from control, LIES, anti-PD-1 blockade, and LIES combined with anti-PD-1 blockade groups. The results showed that the LIES combined with anti-PD-1 blockade group exhibited significantly lower cellular proliferation (Ki67) and enhanced apoptosis (Cleaved Caspase 3) compared to the control, LIES alone, and anti-PD-1 inhibitor alone groups (**Figure 5F**). Additionally, the combination treatment resulted in markedly increased infiltration of CD8^+^ T cells within the tumor microenvironment. These findings indicate that the combination therapy provides superior anti-tumor efficacy compared to monotherapy with either LIES or anti-PD-1 blockade.

### 3.6 LIES upregulates PD-L1 expression by activating the JAK2/STAT3 signaling pathway

**Figure 6:**
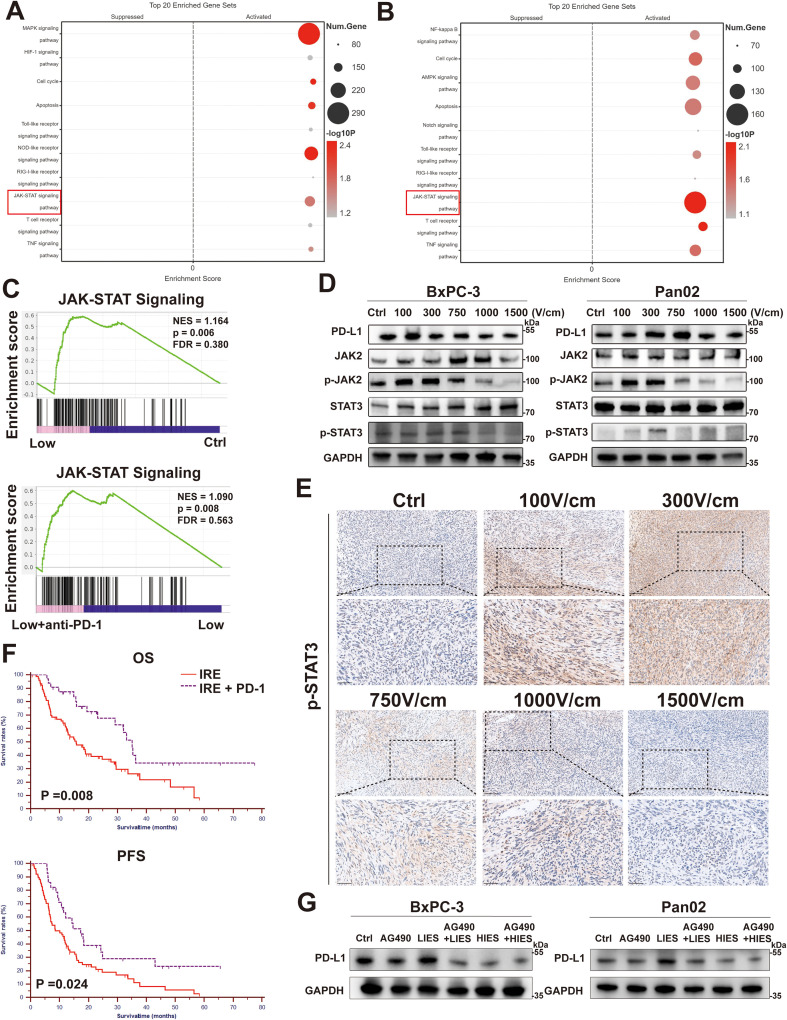
Low-intensity electric stimulation (LIES) upregulates PD-L1 expression by activating the JAK2/STAT3 signaling pathway. **(A)** KEGG pathway analysis of differentially expressed genes in BxPC-3 cells between LIES and control groups revealed the most significant enriched pathways and the p-values. **(B)** Kyoto Encyclopedia of Genes and Genomes (KEGG) pathway analysis of differentially expressed genes in BxPC-3 cells between LIES and LIES + anti-PD-1 groups revealed significant upregulation in the JAK-STAT pathways. **(C)** Gene Ontology (GO) analysis showing upregulation of JAK-STAT pathway in LIES and LIES +anti-PD-1 group. P value is calculated using GO Empirical phenotype-based permutation test, two-sided, and no adjustments were made for multiple comparisons. **(D)** Western blotting analysis of PD-L1, STAT3, p-STAT3, JAK2, and p-JAK2 expression in BxPC-3 and Pan02 cells, under different electric field strengths. **(E)** Representative immunohistochemical staining of CD8^+^, Cleaved Caspase 3, and Ki 67 in orthotopic tumor tissue sections. Low-magnification images, Scale bar, 100 μm; High-magnification images, Scale bar, 50 μm. **(F)** Kaplan-Meier analyses of the correlation between irreversible electroporation (IRE) + anti-PD-1 and IRE group and overall survival or recurrence-free survival in locally advanced pancreatic cancer (LAPC) patients (n=108). **(G)** Inhibition of the JAK2/STAT3 pathway abrogates LIES-induced PD-L1 expression. Representative of western blotting analysis showing the protein levels of PD-L1 in BxPC-3 and Pan02 cells treated with LIES or HIES in the presence or absence of the JAK2 inhibitor AG490 (50 μM). Statistical significance was assessed by log-rank test (P < 0.05).

Based on Kyoto Encyclopedia of Genes and Genomes (KEGG) and Gene Ontology (GO) analysis of RNA-sequencing data, the JAK2/STAT3 signaling pathway was significantly enriched in the LIES group (**Figures 6A–C**). WB analysis further demonstrated increased phosphorylation level of both JAK2 and STAT3 in LIES-treatment samples, indicating activation of this pathway (**Figure 6D**). Moreover, in the LIES group, phosphorylation of JAK2 and STAT3 progressively increased with the enhancement of the electric field strength. In contrast, in the HIES group, the phosphorylation levels of JAK2 and STAT3 showed a diminished increase as the field strength intensified. Notably, this activation was accompanied by a marked upregulation of PD-L1 expression at the protein level, supporting the regulatory role of JAK2/STAT3 signaling in PD-L1 induction by LIES. To further establish a causal link between pathway activation and PD-L1 induction, we employed AG490, a selective JAK2 inhibitor, for functional validation (**Figure 6G**). Our results showed that pharmacological inhibition of JAK2 significantly abolished the LIES-induced phosphorylation of STAT3 and subsequent upregulation of PD-L1 protein levels. Consistently, IHC analysis further validated the increased phosphorylation of STAT3 in the LIES group (**Figure 6E**), collectively confirming that LIES upregulates PD-L1 expression through promoting JAK2 and STAT3 phosphorylation.

### 3.7 Anti-PD-1 blockade prolongs survival after IRE in LAPC patients

From an initial cohort of 1011 pancreatic cancer patients, 108 individuals with locally advanced pancreatic cancer (LAPC) were enrolled and divided into the IRE group (n = 76) and the IRE plus anti-PD-1 blockade group (n = 32, **Supplementary Figure 5A**). Baseline clinicopathological characteristics, including age (p = 0.830), gender (p = 0.059), tumor grade (p = 0.608), and imaging lymph node metastasis (p = 0.207), were well-balanced between the two treatment arms (**Supplementary Figure 5B**).

As shown in **Figure 6F**, patients receiving the combination of IRE ablation and anti-PD-1 blockade demonstrated significantly improved clinical outcomes compared to those treated with IRE ablation alone. Specifically, the median OS was 35.03 months (95% CI: 30.94 - 39.13 months) in the IRE ablation combined with anti-PD-1 blockade group versus 15.77 months (95% CI: 10.23 - 21.31 months) in the IRE ablation alone group (p = 0.008), while the median PFS was 14.33 months (95% CI: 11.19 - 17.43 months) versus 8.53 months (95% CI: 4.05 - 13.02 months, p = 0.024), respectively.

Multivariate Cox regression analysis identified that anti-PD-1 blockade therapy served as a significant independent protective factor for both OS and PFS, whereas advanced age (>60 years), poorer tumor differentiation, and specific adjuvant chemotherapy regimens (e.g., AG/S-1) were associated with increased mortality or disease progression risks (**Supplementary Figures 5C, D**). These findings suggest that the addition of anti-PD-1 blockade therapy to IRE ablation confers substantial survival benefits for patients with LAPC.

## 4 Discussion

**Figure 7:**
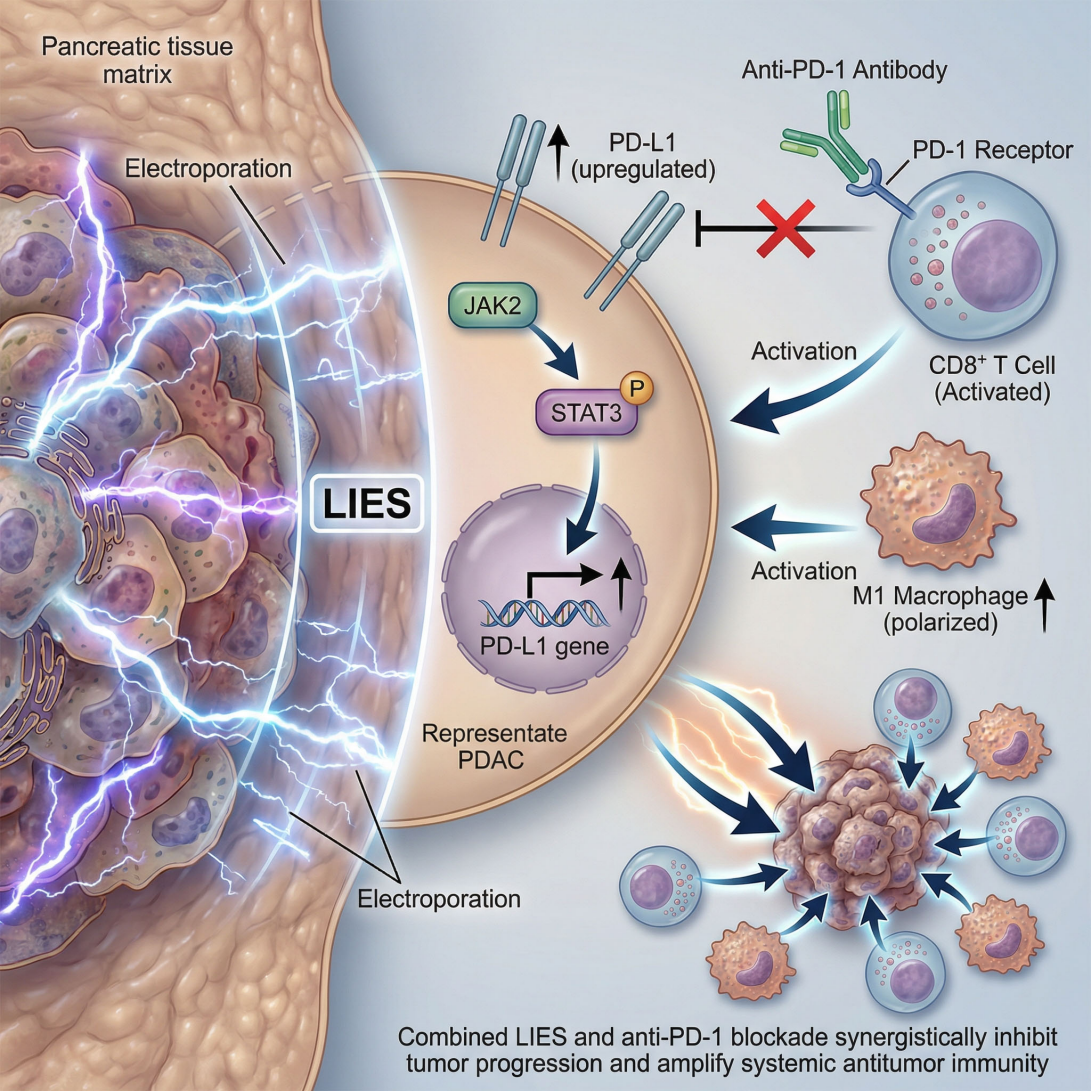
Schematic depiction of the mechanism underlying how low-intensity electric stimulation (LIES) fosters an immune microenvironment in pancreatic cancer, whereas combination with anti-PD-1 blockade impedes malignances progression.

IRE has emerged as a promising therapeutic strategy in LAPC patients who are not eligible for surgical resection. However, some patients still experience local recurrence after IRE (29, 30), highlighting the need for further investigation into the underlying mechanisms to enhance therapeutic efficacy and patient outcomes. The efficacy of IRE is highly contingent upon the administration of electric pulses at strengths above the electroporation threshold. Sub-threshold electric fields lead to the formation of reversible membrane pores, which are rapidly repaired by the cells, preventing cell death. Due to the irregular shape and substantial size of LAPC, certain tumor regions may be subjected solely to LIES, resulting in incomplete ablation and a heightened risk of tumor recurrence (12). However, the immune mechanisms following LIES remain undefined. In this study, we demonstrated that the LIES enhanced tumor cell proliferation and enhanced migratory capacity, while promoting a shift toward a mesenchymal-like phenotype. ScRNA-seq and bulk RNA sequencing analysis of orthotopic murine tumors further revealed upregulation of PD-L1 expression and the enrichment of EMT-related transcriptional signatures in specific tumor cell subsets. In detail, LIES was found to activate the JAK2-STAT3 pathway, leading to increased PD-L1 expression. Anti-PD-1 monotherapy exhibited potent anti-tumor immune activity, effectively reshaping the immune microenvironment that subsequently led to an attenuation of the EMT-like phenotype. This effect was evidenced by increased infiltration of cytotoxic CD8^+^ T lymphocytes and a shift from the M2 to the M1 macrophage polarization. The combination of LIES and anti-PD-1 blockade significantly enhanced CD8^+^ T cell infiltration and further limited the progression of mesenchymal features, resulting in greater tumor cell killing compared to either modality alone (**Figure 7**). Thus, this approach represents a promising therapeutic strategy to enhance the clinical outcomes of IRE treatment for PDAC.

As a novel and promising local ablative therapy, IRE employs short, high-voltage electrical pulses that compromise cell membrane integrity, ultimately leading to apoptotic and necrotic cell death. In most tissues, an electric field strength of 680 V/cm is recognized as the standard threshold for effective IRE (16, 31), leading to irreversible cell death. Below this value, electroporation is typically considered reversible. However, pancreatic tumors often present with irregular shapes, large volumes, and complex peri-tumoral anatomy, which poses a significant challenge to achieving a uniform electric field distribution. In clinical practice, although preoperative planning aims for complete coverage, the inherent steepness of the electric field gradient, combined with factors such as inaccurate electrode placement or the presence of high-impedance structures (e.g., stromal fibrosis), inevitably creates a sublethal zone at the ablation margins. In these peripheral regions, the effective field strength falls below the lethal threshold, subjecting residual tumor cells to insufficient electrical stress. In this study, we formally defined this clinically relevant insufficient stimulation as LIES. The field strength parameters for LIES were specifically selected to mimic the peri-tumoral boundary conditions observed in clinical IRE procedures, ensuring the translational relevance of our findings. Furthermore, previous studies have suggested that regions exposed to LIES may even promote tumor progression (32), although the underlying mechanisms and the biological consequences of this sublethal exposure in the context of the pancreatic tumor microenvironment remain poorly understood. In this study, we further investigated the tumor environment following LIES treatment and identified the necessity for incorporating adjunctive therapeutic strategies to overcome the limitations associated with LIES exposure. PDAC is characterized by a unique immunosuppressive tumor microenvironment, which is largely attributable to sparse immune cell infiltration (33, 34). Following LIES treatment, the expression of PD-L1 was found to be upregulated. Consistent with our findings, previous studies have also reported that IRE can induce increased PD-L1 expression in tumor cells. Khan et al. reported that IRE-induced IFN-γ signaling upregulates PD-L1 expression, leading to a reduction in myeloid-derived suppressor cell (MDSCs) and regulatory T cell (Tregs) and an increase in cytotoxic T cells and neutrophils in a murine model of pancreatic cancer (35). As demonstrated by Erika et al, radiofrequency ablation (RFA) simultaneously initiates antitumor immunity and elicits a PD-L1-mediated adaptive immune resistance, which underlies its transient therapeutic efficacy. Concomitant PD-L1 blockade effectively dismantles this immunosuppressive barrier, converting the short-lived effects of RFA into durable, systemic antitumor responses (36). RFA primes cytotoxic T cells yet upregulates PD-L1 to exhaust them; adding PD-1 blockade reinvigorates effector function, curtails Tregs, and converts fleeting effects into durable systemic immunity (37). CD8^+^ T cells have gained considerable focus in the context of anti-tumor immunity due to their potent ability to eliminate tumor cells. This study demonstrated that LIES alone was not sufficient to enhance CD8^+^ T cell infiltration. In contrast, HIES was capable of promoting CD8^+^ T cell infiltration on its own. Additionally, LIES could upregulate PD-L1 expression through the JAK2-STAT3 signaling pathway, and when combined with anti-PD-1 blockade, it further facilitated increased CD8^+^ T cell infiltration. CD8^+^ T cells target tumors through direct cytotoxicity by releasing perforin and granzymes, secreting cytokines such as IFN-γ and IL-2, and activating the death receptor pathway, such as Fas/FasL-mediated apoptosis (38, 39). Additionally, novel subsets of CD8^+^ T cells, including MHC Ib-restricted cells and stem-like cells, exhibit broad-spectrum anti-tumor and anti-exhaustion characteristics (40, 41). Strategies aimed at reversing T cell exhaustion and utilizing memory T cells, such as tissue-resident memory T cells and central memory T cells, to maintain sustained immune responses have emerged as critical approaches to improve anti-tumor efficacy (42). The findings of this study are consistent with previous studies. The research by Jun Zhao et al. demonstrated that IRE improved the efficacy of anti-PD-1 by inducing immunogenic cell death, activating dendritic cells, and remodeling the tumor microenvironment, which enhances CD8^+^ T cell recruitment and activation, thus overcoming the resistance of PDAC to immune checkpoint inhibitors (15). Another study showed that, compared to dual or single treatment regimens, the combination of IRE, anti-PD-1blockade, and TGF-β1receptor inhibition significantly enhances T cell responses and induces a shift of tumor-associated neutrophils from the N2 phenotype to the N1 phenotype (43). Our investigations also showed that LIES and HIES combined treatment with anti-PD-1 inhibitors resulted in significantly higher levels of mature DCs and effector memory T cells in each group compared to the corresponding groups without anti-PD-1 inhibitors. Furthermore, the synergistic effect of anti-PD-1 inhibitors was more pronounced with increasing electric field intensity. Jayanth et al. reported that the combination of IRE and intratumoral CD40 antibodies inhibits orthotopic PDAC by activating DCs and enhancing T cell responses to novel antigens (22). Weichen Xu et al. demonstrated that IRE combined with a macrophage-based proteolipid vesicle coencapsulating the CCR2 antagonist PF and gemcitabine significantly increased CD8^+^ T cell infiltration and induced long-lasting immune memory, characterized by an increased proportion of effector memory T cells (44). These results are consistent with our findings.

Tumor-associated macrophages (TAMs) play a key role in PDAC progression by polarizing into M1 or M2 phenotypes (45, 46). M1 macrophages suppress tumors through proinflammatory mediators, while M2 macrophages promote tumor growth by secreting anti-inflammatory cytokines (47). M2 polarization also contributes to desmoplastic stroma formation, aiding immune escape (48, 49). Clinically, increased M2 macrophage levels correlate with greater PDAC malignancy (50, 51). In our previous study, we demonstrated that HIES promoted M1 macrophage (52). However, the impact of LIES on macrophage polarization remains unclear, and it is yet to be determined whether the combination of anti-PD-1 blockade and LIES can reverse M2 polarization. We observed that LIES treatment was associated with a shift toward a mesenchymal-like phenotype and facilitated a microenvironment conducive to M2-like macrophage polarization. Bo Sun et al. demonstrated that iRFA induces an immunosuppressive tumor microenvironment characterized by an increased infiltration of M2 polarized TAMs and other immunosuppressive cells (53). Following the anti-PD-1 blockade, the EMT process induced by LIES was mitigated. In glioblastoma, anti-PD-1 therapy eliminates PD-1-expressing immunosuppressive macrophages and promotes the M1 phenotype (54). Radiotherapy combined with anti-PD-1 and anti-TIGIT antibodies promotes the polarization of M1 macrophages while inhibiting M2 macrophage polarization, which results in reduced CD8^+^ T cell activation and diminished anti-tumor efficacy (55). In summary, anti-PD-1 blockade can alleviate LIES-induced M2 polarization and enhance M1 polarization.

This study has several limitations. First, the non-resective nature of IRE treatment made it challenging to obtain human pancreatic cancer samples. Although a murine orthotopic model was used as a surrogate, differences in immune composition and the tumor microenvironment underscore the need for further validation in human specimens. Second, the limited availability of recurrent tumor samples following LIES treatment restricted our ability to directly investigate recurrence mechanisms. Future research should integrate clinical patient cohorts, liquid biopsy analyses, and patient-derived organoid models to better understand the dynamics of the tumor microenvironment and the mechanisms underlying recurrence after IRE, to optimize its therapeutic efficacy in pancreatic cancer. Third, although we utilized Pan02 cells to establish tumor models, which closely mimic the pathogenesis of human PDAC, it remains unclear whether similar immune cell alterations occur in human patients following IRE treatment.

## 5 Conclusions

This study elucidated that LIES is associated with tumor progression by activating the JAK2/STAT3 signaling axis, which correlates with the upregulation of PD-L1 expression and the promotion of an M2-like macrophage phenotype. Anti-PD-1 blockade can reshape the tumor microenvironment, characterized by upregulating CD8^+^ T cell expression and a rebalancing toward M1-like polarization. Moreover, the integration of anti-PD-1 blockade with IRE potentially overcomes the limitations of incomplete ablation within the LIES, thereby improving oncological outcomes.

## Data Availability

The original contributions presented in the study are included in the article/**Supplementary Material**. Further inquiries can be directed to the corresponding authors.

## Supplementary material

Supplementary Figure 1 Combined low-intensity electric stimulation (LIES) and anti-PD-1 blockade treatment suppresses PD-L1 and epithelial-mesenchymal transition (EMT) expression, related to **Figure 3**. **(A)** Immunofluorescence (IHF) analysis in tumor sections from orthotopic pancreatic tumors. Nuclei were stained with DAPI (blue), tumor cells with panCK (cyan), and M2 macrophages with CD206 (yellow) fluorescence. Merged images were displayed in the leftmost column. Scale bar, 50 μm. Data in **(B)** are shown as relative CD206 expression. **(C)** Single-cell RNA sequencing analysis of orthotopic tumors. UMAP plots showed the clustering of single cells based on different annotation schemes: (upper left) Seurat clusters, (upper right) cell type annotations, and (lower panel) original sample identity. Cells are color-coded according to their assigned clusters, cell types, and sample identities. **(D)** Dot plot displayed the expression of key marker genes across different cell identities. Each dot represents a gene, with the size indicating the percentage of cells expressing the gene and the color representing the average expression level. Data in **(B)** are presented as mean ± SD (n = 10). *p < 0.05; **p < 0.01; ***p < 0.001; ****p < 0.0001, significant difference compared with the control (one-way ANOVA, Dunnett’s test).

Supplementary Figure 2 Single-cell transcriptomic profiling of murine orthotopic pancreatic tumors. **(A)** UMAP plots showed the clustering of single cells based on different annotation schemes: (left) Seurat clusters, (middle) cell type annotations, and (right) original sample identity. Cells are color-coded according to their assigned clusters, cell types, and sample identities. **(B)** Dot plot displayed the expression of key marker genes across different cell identities. Each dot represents a gene, with the size indicating the percentage of cells expressing the gene and the color representing the average expression level.

Supplementary Figure 3 Combined low-intensity electric stimulation (LIES) and anti-PD-1 blockade treatment enhances CD8^+^ T cell infiltration, related to **Figure 4**. **(A, C, D)** Representative plots and percentages of mature dendritic cells (DCs) and effector memory T cells in orthotopic pancreatic tumors under different electric field strengths and anti-PD-1 blockade treatment (n=3). **(B, E, F)** Representative plots and percentages of CD4^+^ T cells, CD8^+^ T cells, and effector memory T cells in spleens of mice under different electric field strengths and anti-PD-1 blockade treatment (n=3). *p < 0.05; **p < 0.01; ***p < 0.001; ****p < 0.0001, significant difference compared with the control or without anti-PD-1 blockade treatment (one-way ANOVA, Dunnett’s test).

Supplementary Figure 4 Effects of electric field strength and anti-PD-1 blockade on splenic activated dendritic cells (DCs) in mice, related to **Figure 4**. **(A)** Representative plots and percentages of dendritic cells in the spleen (n=3).

Supplementary Figure 5 Patient cohort selection, baseline clinical characteristics, and multifactorial prognostic analyses for overall survival (OS) and progression-free survival (PFS). **(A)** Flowchart of the retrospective cohort study. **(B)** Distribution of baseline clinical and pathological characteristics between treatment groups. **(C)** Multivariate Cox proportional hazards model for OS. **(D)** Multivariate Cox proportional hazards model for PFS.

Supplementary Table 1 Antibodies used for investigation.

Supplementary Table 2 Primers for real-time PCR.

## Glossary

PDAC: pancreatic ductal adenocarcinoma
LAPC: locally advanced pancreatic cancer
IRE: irreversible electroporation
TME: tumor microenvironment
RE: reversible electroporation
iRFA: insufficient radiofrequency ablation
LIES: low-intensity electric stimulation
NK: natural killer
EMT: epithelial-mesenchymal transition
HIES: high-intensity electric stimulation
PBS: phosphate-buffered saline
RPIM 1640: Rosewell Park Memorial Institute 1640 medium
DMEM: Dulbecco’s modified Eagle medium
GFP: green fluorescent protein
CCK-8: Cell Counting Kit-8
WB: western blotting
SDS-PAGE: sodium dodecyl sulfate-polyacrylamide gel electrophoresis
PVDF: polyvinylidene difluoride
IHC: immunohistochemistry
IHF: immunohistofluorescence
DAB: 3,3'-diaminobenzidine
HRP: horseradish peroxidase
cDNA: complementary DNA
qPCR: quantitative reverse transcription polymerase chain reaction
scRNA-seq: single-cell RNA sequencing
PCA: Principal Component Analysis
UMAP: Uniform Manifold Approximation and Projection
DCs: endritic cells
CTLs: cytotoxic T lymphocytes
ETLs: exhausted T lymphocytes
OS: overall survival
KEGG: Kyoto Encyclopedia of Genes and Genomes
GO: Gene Ontology
PFS: progression-free survival
MDSCs: myeloid-derived suppressor cell
Tregs: regulatory T cell
RFA: radiofrequency ablation
TAMs: tumor-associated macrophages.
