# Supplementary figures and images for: Anti-PD-1 blockade reverses low-intensity electric stimulation-driven pancreatic cancer progression
Source: Front Immunol. 2026 May 19;17:1793161. doi: 10.3389/fimmu.2026.1793161 (PMC13226209; doi:10.3389/fimmu.2026.1793161)

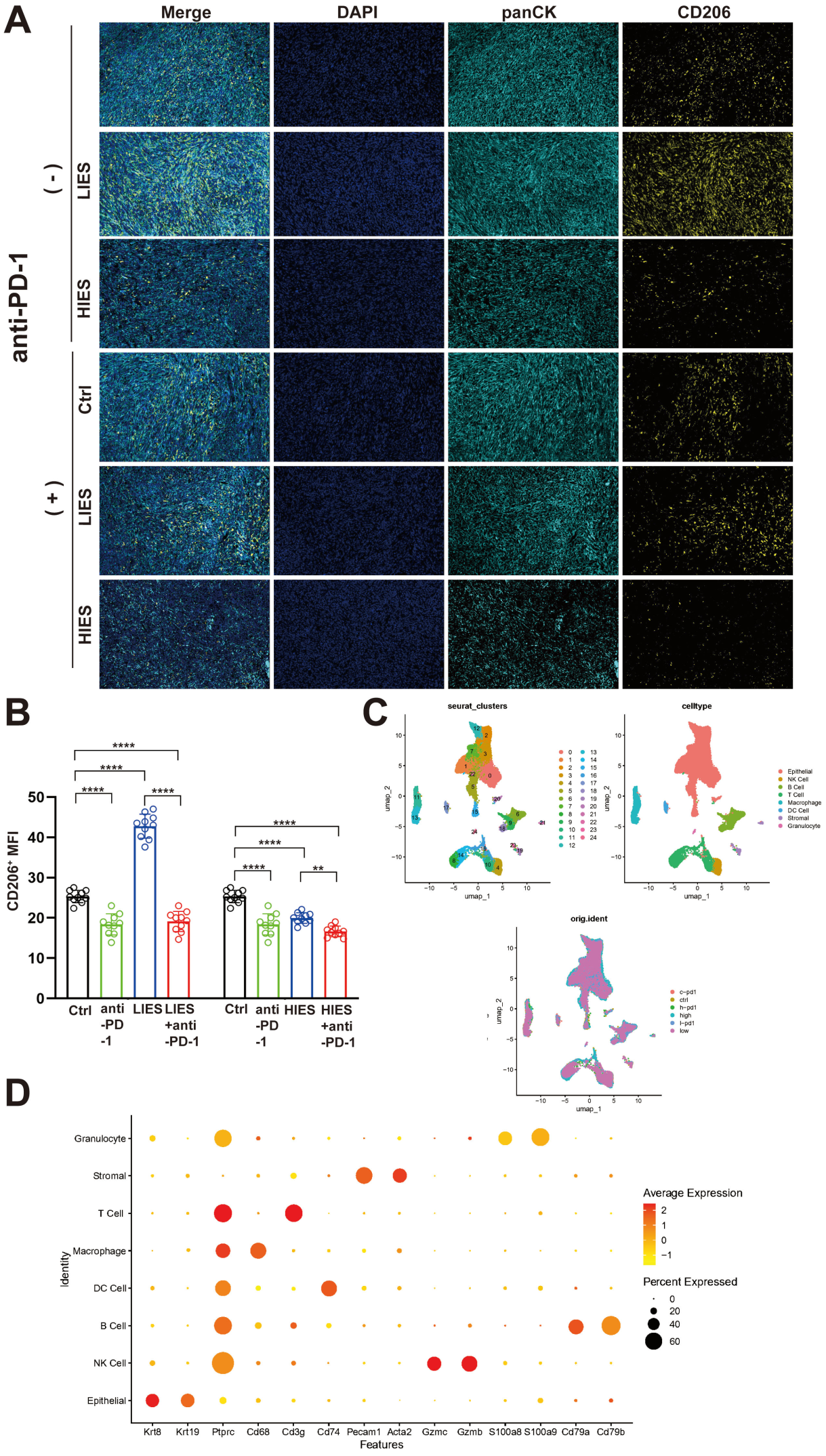

Supplement: Supplementary Figure 1 — Combined low-intensity electric stimulation (LIES) and anti-PD-1 blockade treatment suppresses PD-L1 and epithelial-mesenchymal transition (EMT) expression, related to Figure 3. (A) Immunofluorescence (IHF) analysis in tumor sections from orthotopic pancreatic tumors. Nuclei were stained with DAPI (blue), tumor cells with panCK (cyan), and M2 macrophages with CD206 (yellow) fluorescence. Merged images were displayed in the leftmost column. Scale bar, 50 μm. Data in (B) are shown as relative CD206 expression. (C) Single-cell RNA sequencing analysis of orthotopic tumors. UMAP plots showed the clustering of single cells based on different annotation schemes: (upper left) Seurat clusters, (upper right) cell type annotations, and (lower panel) original sample identity. Cells are color-coded according to their assigned clusters, cell types, and sample identities. (D) Dot plot displayed the expression of key marker genes across different cell identities. Each dot represents a gene, with the size indicating the percentage of cells expressing the gene and the color representing the average expression level. Data in (B) are presented as mean ± SD (n = 10). *p < 0.05; **p < 0.01; ***p < 0.001; ****p < 0.0001, significant difference compared with the control (one-way ANOVA, Dunnett’s test). [file Image1.tif]

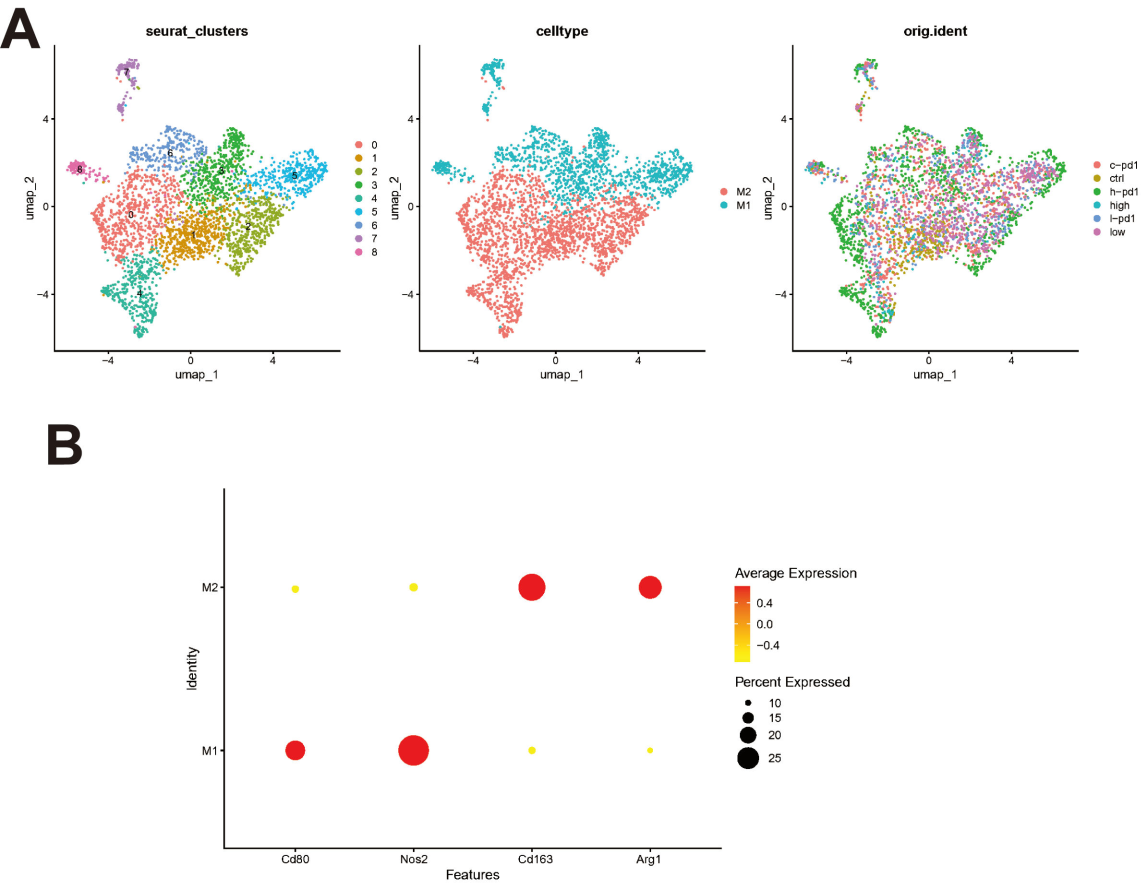

Supplement: Supplementary Figure 2 — Single-cell transcriptomic profiling of murine orthotopic pancreatic tumors. (A) UMAP plots showed the clustering of single cells based on different annotation schemes: (left) Seurat clusters, (middle) cell type annotations, and (right) original sample identity. Cells are color-coded according to their assigned clusters, cell types, and sample identities. (B) Dot plot displayed the expression of key marker genes across different cell identities. Each dot represents a gene, with the size indicating the percentage of cells expressing the gene and the color representing the average expression level. [file Image2.tif]

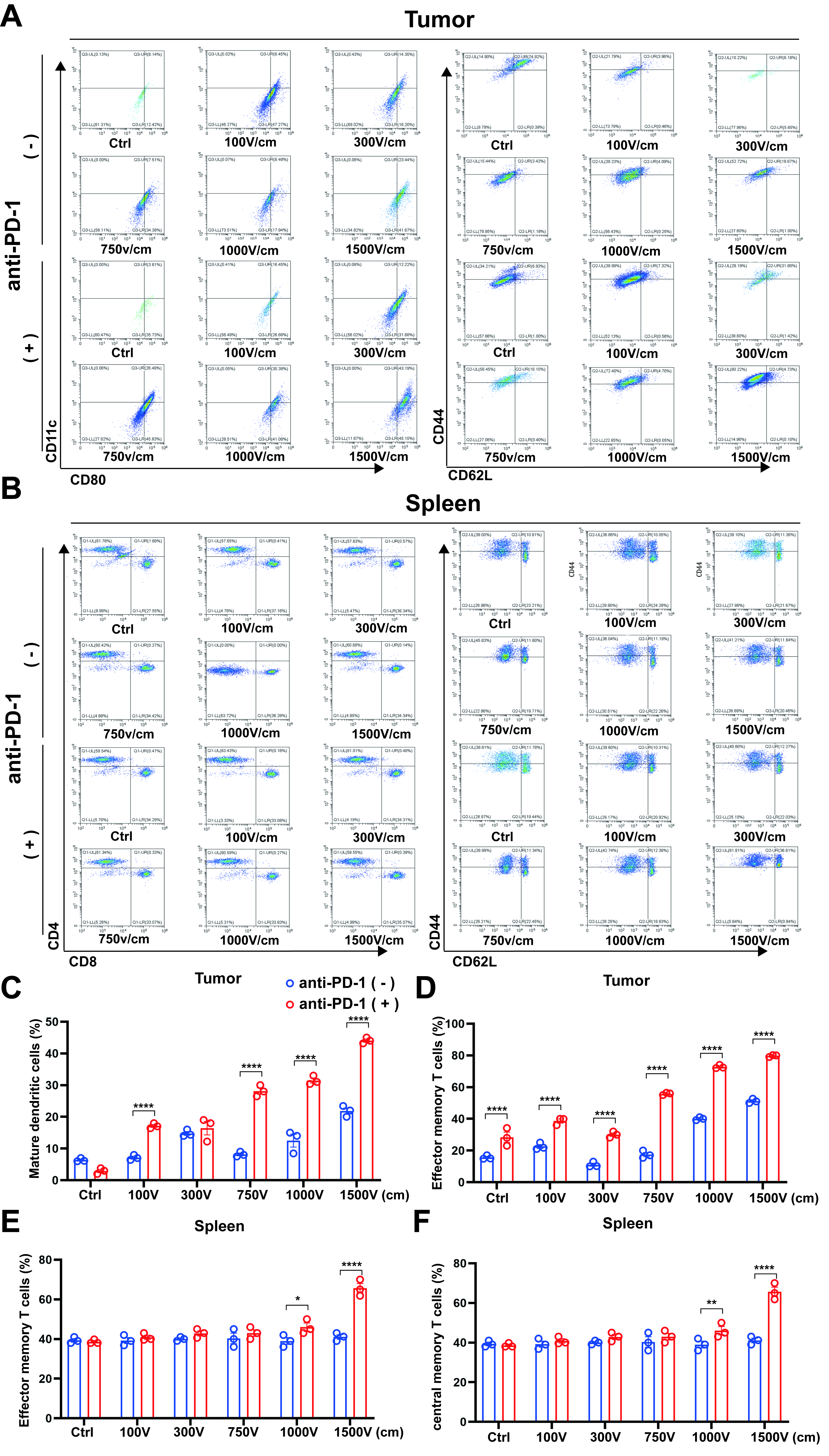

Supplement: Supplementary Figure 3 — Combined low-intensity electric stimulation (LIES) and anti-PD-1 blockade treatment enhances CD8+ T cell infiltration, related to Figure 4. (A, C, D) Representative plots and percentages of mature dendritic cells (DCs) and effector memory T cells in orthotopic pancreatic tumors under different electric field strengths and anti-PD-1 blockade treatment (n=3). (B, E, F) Representative plots and percentages of CD4+ T cells, CD8+ T cells, and effector memory T cells in spleens of mice under different electric field strengths and anti-PD-1 blockade treatment (n=3). *p < 0.05; **p < 0.01; ***p < 0.001; ****p < 0.0001, significant difference compared with the control or without anti-PD-1 blockade treatment (one-way ANOVA, Dunnett’s test). [file Image3.tif]

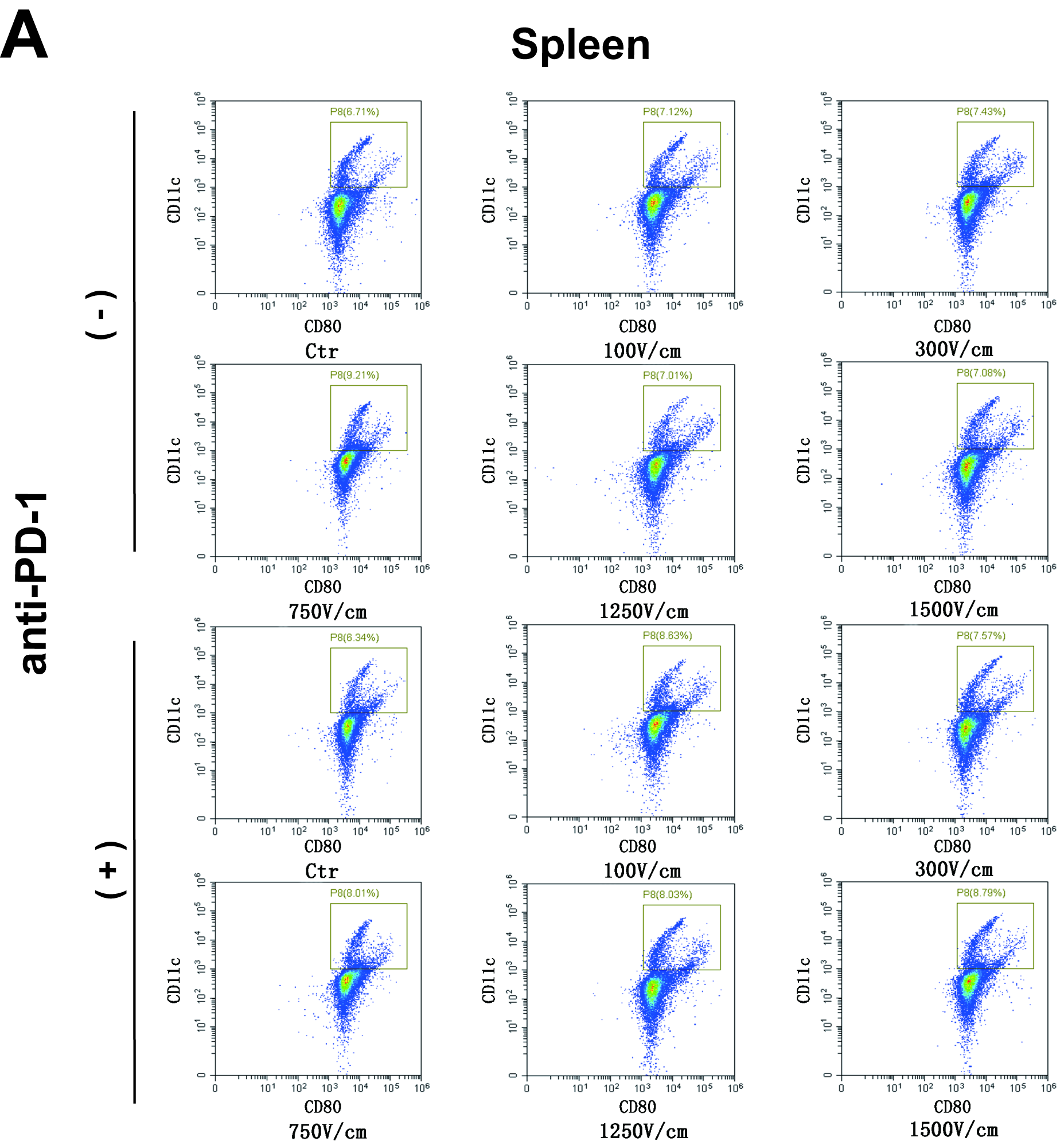

Supplement: Supplementary Figure 4 — Effects of electric field strength and anti-PD-1 blockade on splenic activated dendritic cells (DCs) in mice, related to Figure 4. (A) Representative plots and percentages of dendritic cells in the spleen (n=3). [file Image4.tif]

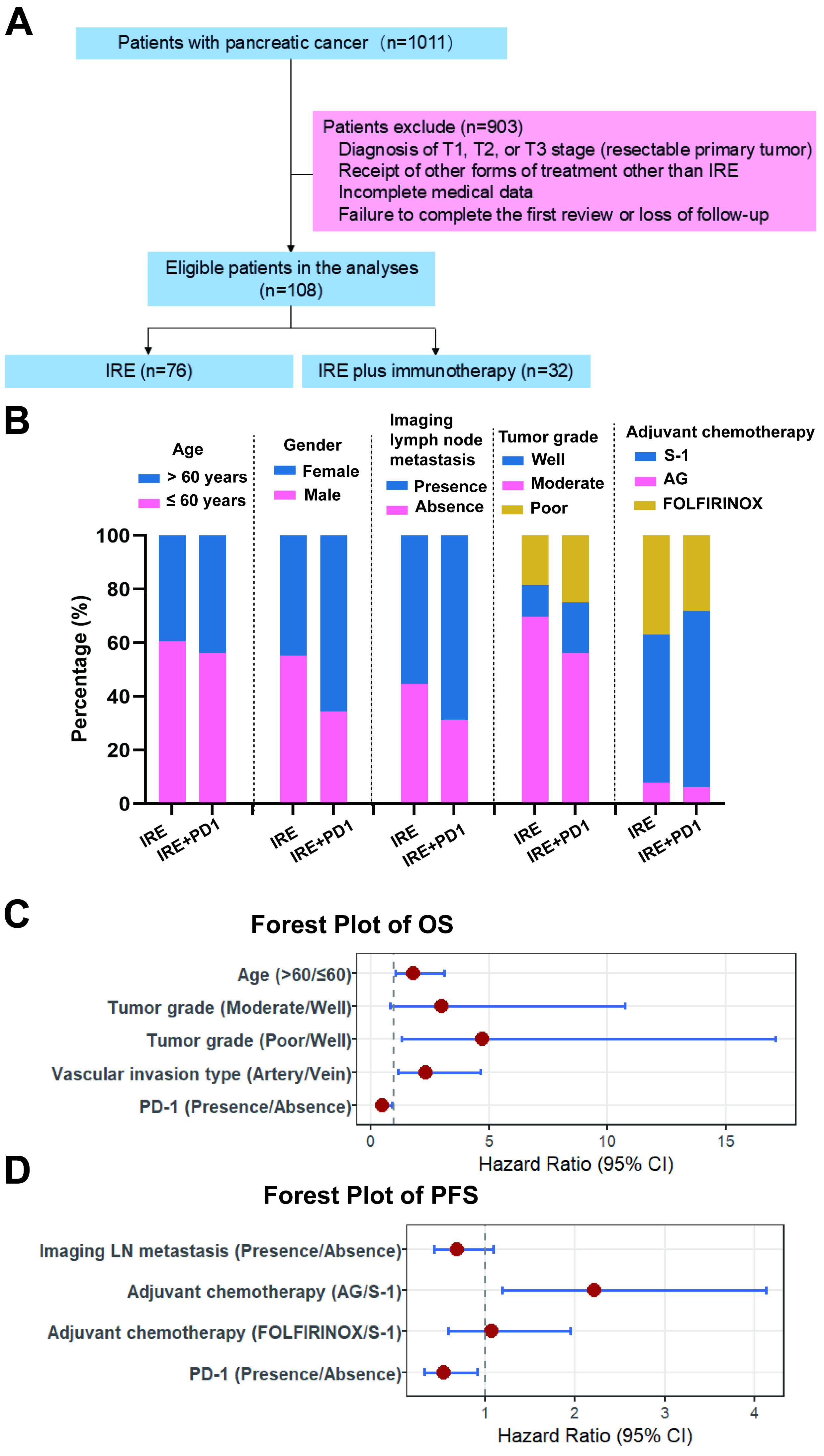

Supplement: Supplementary Figure 5 — Patient cohort selection, baseline clinical characteristics, and multifactorial prognostic analyses for overall survival (OS) and progression-free survival (PFS). (A) Flowchart of the retrospective cohort study. (B) Distribution of baseline clinical and pathological characteristics between treatment groups. (C) Multivariate Cox proportional hazards model for OS. (D) Multivariate Cox proportional hazards model for PFS. [file Image5.tif]

Figure 2C

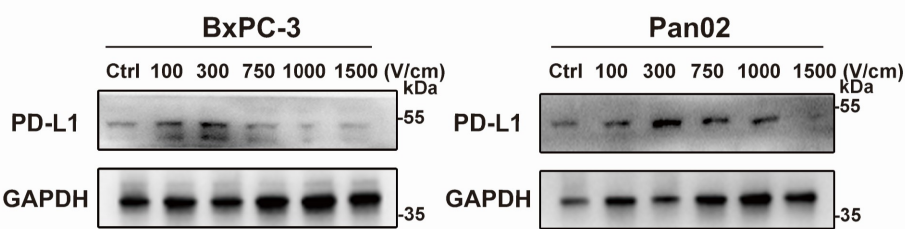

Seperated gel

marker: MP102-02, Vazyme

BxPC-3

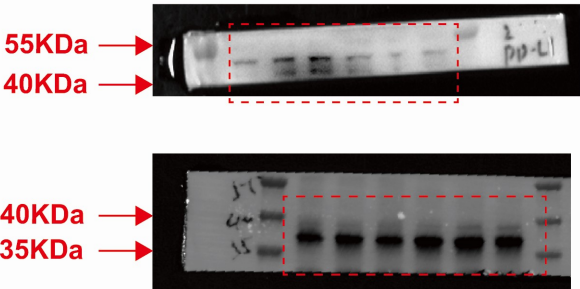

Pan02

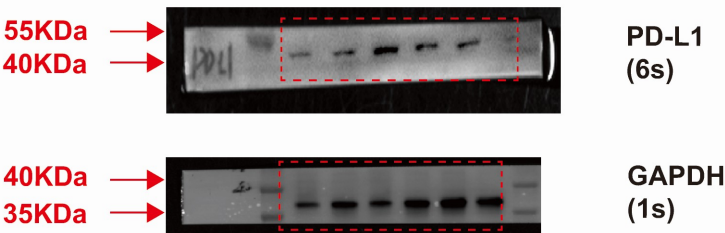

Supplement: Supplementary file 8 [file DataSheet1.pdf]

Figure 2E

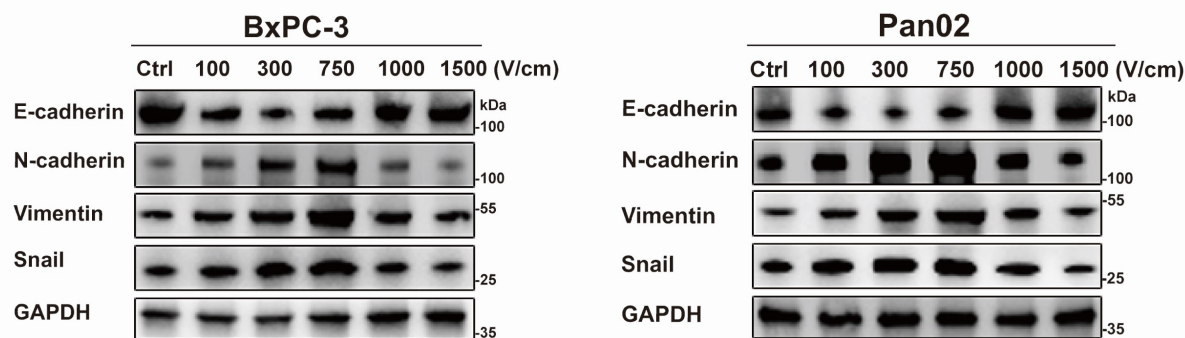

Seperated gel  
marker: MP102-02, Vazyme

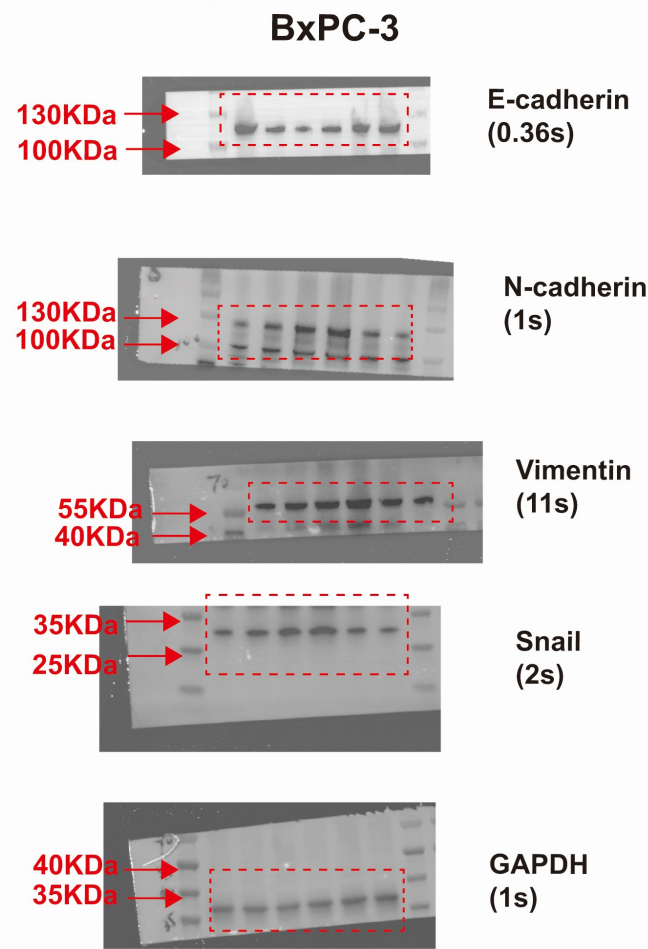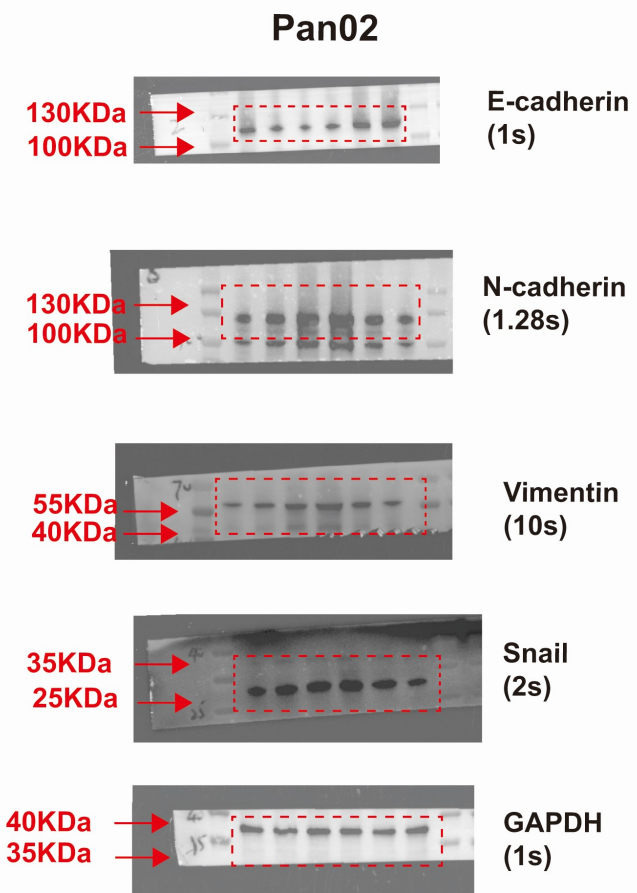

Supplement: Supplementary file 9 [file DataSheet2.pdf]
