# Supplementary material for: Anti-PD-1 blockade reverses low-intensity electric stimulation-driven pancreatic cancer progression
Source: Front Immunol. 2026 May 19;17:1793161. doi: 10.3389/fimmu.2026.1793161 (PMC13226209; doi:10.3389/fimmu.2026.1793161)
Supplement: Supplementary Table 1 — Antibodies used for investigation. [file Table1.docx]

Supplementary Table 1: Antibodies used for investigation

| Antibody | Source | Identifier |
| --- | --- | --- |
| PE anti-mouse CD274 | Biolegend | 124307 |
| FITC anti-mouse CD80 | Biolegend | 104705 |
| PE anti-mouse CD62L | Biolegend | 104417 |
| FITC anti-mouse CD44 | Biolegend | 103006 |
| Brilliant Violet 605 anti-mouse CD11c | Biolegend | 117334 |
| Anti-CD4 Rabbit monoclonal | Abcam | Ab183685 |
| Anti-CD8 Rabbit monoclonal | Abcam | Ab209775 |
| FITC anti-mouse CD206 | Biolegend | 141703 |
| CD206 Rabbit mAb | Cell Signaling Technology | 24595T |
| PE anti-mouse CD16/32 | Biolegend | 101307 |
| PD-L1 Polyclonal antibody | Proteintech | 28076-1-AP |
| Ki-67 Polyclonal antibody | Abcam | Ab16667 |
| GAPDH Rabbit mAb | Proteintech | 10494-1-AP |
| Cleaved Caspase3 | Proteintech | 25128-1-AP |
| Anti-pan Cytokeratin | Abcam | Ab264485 |
| Vimentin Monoclonal antibody | Proteintech | 60330-1-Ig |
| N-cadherin Polyclonal antibody | Proteintech | 22018-1-AP |
| E-cadherin Polyclonal antibody | Proteintech | 20874-1-AP |
| SNAIL2 Polyclonal antibody | ABclonal | A11794 |
| STAT3 Polyclonal antibody | Proteintech | 10253-2-AP |
| Phopho-STAT3 Polyclonal antibody | Proteintech | 60479-1-Ig |
| JAK2 Rabbit mAb | Cell Signaling Technology | 3230T |
| Phospho-JAK2 Rabbit mAb | Cell Signaling Technology | 4406T |
| Anti-rabbit IgG HRP-linked Antibody | ABclonal | AS003 |
| Anti-mouse IgG HRP-linked Antibody | Proteintech | SA00001-1 |
