# Supplementary material for: Anti-PD-1 blockade reverses low-intensity electric stimulation-driven pancreatic cancer progression
Source: Front Immunol. 2026 May 19;17:1793161. doi: 10.3389/fimmu.2026.1793161 (PMC13226209; doi:10.3389/fimmu.2026.1793161)
Supplement: Supplementary Table 2 — Primers for real-time PCR. [file Table2.docx]

Supplementary Table 2: Primers for real-time PCR

| Gene |  | Sequences |
| --- | --- | --- |
| Human GAPDH | Forward | TGCACCACCAACTGCTTAGC |
|  | Reverse | GGCATGGACTGTGGTCATGAG |
| Human PDL1 | Forward | AAGTCCTGAGTGGTAAGA |
|  | Reverse | TTAGTTGTTGTGTTGATTCTC |
| Mouse GAPDH | Forward | GGCACAGTCAAGGCTGAGAATG |
|  | Reverse | ATGGTGGTGAAGACGCCAGTA |
| Mouse PDL1 | Forward | CACAGAACAGGACTCACT |
|  | Reverse | GTATCTTCAACGCCACATT |
