# Supplementary material for: Anti-PD-1 blockade reverses low-intensity electric stimulation-driven pancreatic cancer progression
Source: Front Immunol. 2026 May 19;17:1793161. doi: 10.3389/fimmu.2026.1793161 (PMC13226209; doi:10.3389/fimmu.2026.1793161)

Figure 6D

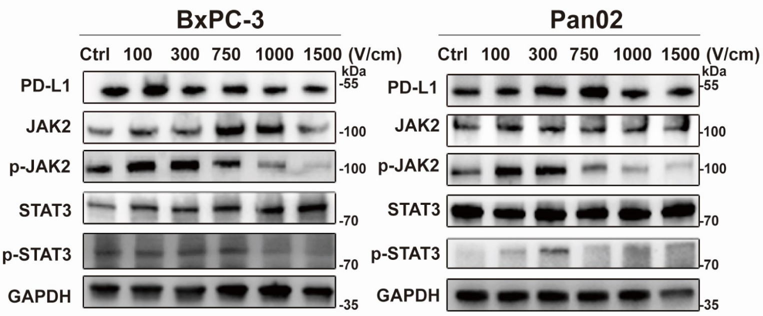

Seperated gel

marker: MP102-02, Vazyme

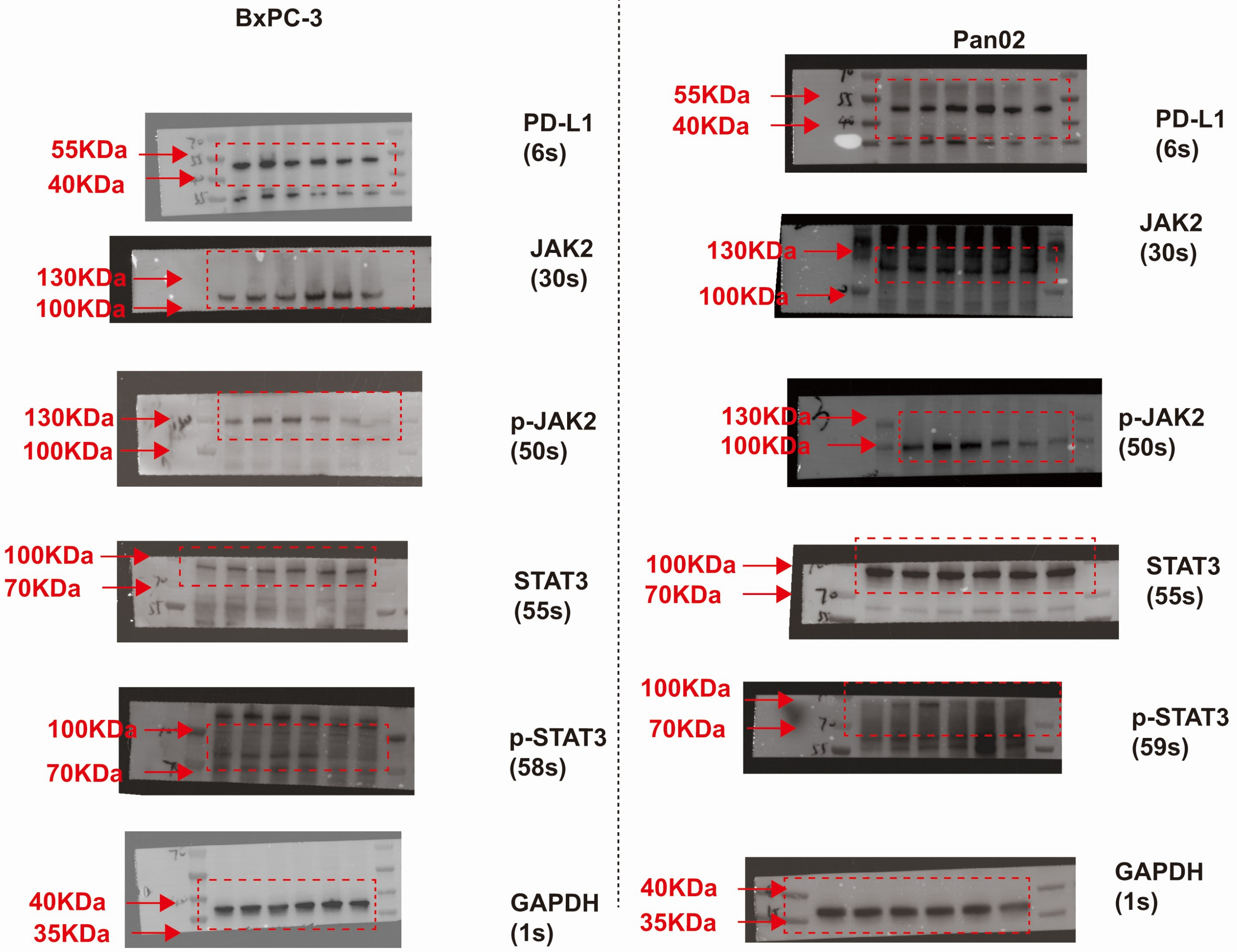

Supplement: Supplementary file 10 [file DataSheet3.pdf]
